# Multiomics characterization of acute child illness and mortality in Africa and South Asia

**DOI:** 10.1038/s41467-026-69754-w

**Published:** 2026-04-13

**Authors:** Camilo A. Espinosa, James M. Njunge, Kirkby D. Tickell, Abdoulaye Hama Diallo, Abu Sadat Mohammad Sayeem Bin Shahid, Md Amran Gazi, Zaubina Kazi, Emily Yoshioka, Caroline Tigoi, Moses Mburu, Moses Ngari, Narshion Ngao, Elisha Omer, Wilson Gumbi, Bonface M. Gichuki, Anna Mitchel, Jessica Williams, Joseph Gogain, Nebojsa Janjic, Rupasri Mandal, Benjamin Jenkins, Hilary P. Browne, Yan Shao, Timothy Rozday, Mark D. Stares, Nicholas J. R. Dawson, Eloise Berson, Alan Chang, Yeasul Kim, Samson J. Mataraso, Chi-Hung Shu, Thanaphong Phongpreecha, Lei Xue, Ali Saleem, Benson Singa, Tahmeed Ahmed, Wieger P. Voskuijl, David S. Wishart, Eric R. Houpt, Jie Liu, Asad Ali, Ezekiel Mupere, Mohammod Jobayer Chisti, Robert H. J. Bandsma, Trevor D. Lawley, Albert Koulman, Christina L. Lancioni, Nima Aghaeepour, James A. Berkley, Judd L. Walson

**Affiliations:** 1 Department of Anesthesiology, Perioperative and Pain Medicine, StanfordStanford University School of Medicine, Stanford, CA USA; 2 Department of Pediatrics, Stanford University School of Medicine, Stanford, CA USA; 3 Department of Biomedical Informatics, Stanford University School of Medicine, Stanford, CA USA; 4 The Childhood Acute Illness and Nutrition Network, Nairobi, Kenya; 5 KEMRI-Wellcome Trust Research Programme, Kilifi, Kenya; 6 Global Health and Epidemiology, University of Washington, Seattle, WA USA; 7 Department of Public Health, Faculty of Health Sciences, University of Ouagadougou, Ouagadougou, Burkina Faso; 8 Nutrition and Clinical Services Division, International Centre for Diarrhoeal Disease Research, Bangladesh (icddrb), Dhaka, Bangladesh; 9 Department of Pediatrics and Child Health, Aga Khan University Hospital, Karachi, Pakistan; 10 Wellcome Sanger Institute, Hinxton, UK; 11 Standard BioTools, Inc., Standard BioTools, Inc., Boulder, CO USA; 12 Department of Biological Sciences, University of Alberta, Edmonton, AB Canada; 13 Core Metabolomics and Lipidomics Laboratory, Metabolic Research Laboratories, Institute of Metabolic Science, University of Cambridge, Cambridge, UK; 14 Department of Pathology, Stanford University School of Medicine, Stanford, CA USA; 15 Kenya Medical Research Institute, Nairobi, Kenya; 16 Amsterdam Institute for Global Child Health, Emma Children’s hospital, Amsterdam UMC, University of Amsterdam, Amsterdam, the Netherlands; 17 Department of Global Health, Amsterdam Centre for Global Health and Development, Amsterdam UMC, University of Amsterdam, Amsterdam, the Netherlands; 18 Department of Paediatrics and Child Health, Kamuzu University of Health Sciences, Blantyre, Malawi; 19 Division of Infectious Diseases and International Health, University of Virginia, Charlottesville, VA USA; 20 School of Public Health, Qingdao University, Qingdao, China; 21 Department of Paediatrics and Child Health, College of Health Sciences, Makerere University, Kampala, Uganda; 22 Centre for Global Child Health, The Hospital for Sick Children, Toronto, ON Canada; 23 Department of Biomedical Sciences, University of Malawi College of Medicine, Blantyre, Malawi; 24 NIHR BRC Nutritional Biomarker Laboratory, University of Cambridge, Cambridge, UK; 25 Department of Pediatrics, Oregon Health and Science University, Portland, OR USA; 26 Center for Tropical Medicine and Global Health, University of Oxford, Oxford, UK; 27 Department of International Health, Johns Hopkins Bloomberg School of Public Health, Baltimore, MD USA

**Keywords:** Predictive markers, Malnutrition, Paediatric research, Machine learning

## Abstract

Childhood illnesses from infectious diseases in low- and middle-income countries contribute substantially to the global under-five mortality. Many hospitalized children experience incomplete recovery, readmission, and post-discharge mortality despite guideline-directed care. However, targeted interventions remain elusive due to limited understanding of underlying mechanisms. In this work, we employ multiomic profiling and multivariate modeling to investigate biological drivers of inpatient and post-discharge mortality in 3,101 acutely ill children across nine sites in sub-Saharan Africa and South Asia. In a nested case-cohort (*N* = 1008), we generate plasma proteomics, serum metabolomics and lipidomics, stool metagenomics, and fecal pathogen data at admission and discharge. Additionally, we profile 270 geographically matched community children for biological baselines. We identify a generalizable mortality signature marked by immune, inflammatory, and metabolic dysregulation with gut dysbiosis. We show that mortality-associated signals persist from admission through discharge, indicating unresolved disease and that malnourished children show greater baseline perturbations, explaining elevated risk. We also find some children with low clinical severity display high predicted mortality risk from targeted biomarkers. Finally, we distill predictive models to a clinically feasible biomarker panel and validate our findings in an independent cohort (*N* = 100). By linking inpatient and post-discharge mortality to specific biological mechanisms, our findings highlight why current care can fail and demonstrate how biomarker-guided risk stratification can identify vulnerable children currently missed by clinical assessments, enabling targeted interventions to reduce mortality in low- and middle-income countries.

## Abstract (web summary)

Many hospitalised children with acute illness in low- and middle-income countries experience incomplete recovery, readmission, and post-discharge mortality despite guideline-directed care. Here the authors report multiomic profiling to investigate biological drivers of hospital in-patient and post-discharge mortality in 3,101 acutely ill children across nine sites in sub-Saharan Africa and South Asia.

## Introduction

Child mortality remains unacceptably high in low- and middle-income countries (LMICs), where more than 80% of global deaths of children under the age of five occur each year^1^. Just in 2019, approximately 5.2 million children under five died globally, primarily in sub-Saharan Africa and South Asia^2^. Mortality in hospitalized children in LMICs is generally related to common infectious illnesses such as pneumonia or gastroenteritis. These children also often display unstable health trajectories after hospitalization, characterized by incomplete recovery, readmission and significant levels of post-discharge mortality despite guideline-directed inpatient care^3–5^. Thus, children discharged from hospitals still face an 8-fold higher risk of death compared to their peers in the community^6^. Hospital admission and re-admission in LMICs are often influenced by costs, accessibility, and the high prevalence of comorbidities, including malnutrition, which impact disease progression and complicate clinical care^7–10^.

New interventions to reduce child mortality in LMICs often show limited success due to a variety of factors. Many interventions originate from high-income settings and may not adequately match the biological and environmental characteristics of children in LMICs^11,12^. While risk scores and protocols based on clinical signs have been developed, these are often under-implemented or have been shown to be ineffective at scale^13,14^. Importantly, many proposed interventions do not account for the complex intersection of health and social issues common in children in these settings, such as co-occurring illnesses, malnutrition, chronic conditions and poverty^15,16^. Interventions to reduce child mortality in LMICs must address the multiple factors that drive child acute illness and pathways to mortality to succeed^3,11,16^.

The emergence of high-throughput technologies for simultaneously examining multiple layers of human biology has led to a revolution in precision medicine^17–20^. By gathering detailed omics data, researchers can identify new biomarkers and develop hypotheses about underlying biological mechanisms of disease^21,22^. Furthermore, the integration of social, clinical, and multiomic data has the potential to clarify the effects of complex factors on disease mechanisms, with therapeutic implications for LMICs^22^. Multiomic profiling studies characterizing child acute illness in LMICs have been limited, with previous work focusing on specific conditions such as complicated severe malnutrition^10^ and sepsis^12,23,24^. Even fewer studies have specifically focused on post-discharge mortality using omics profiling^23,25–27^. Thus, there is an urgent need for multiomic studies in LMICs that consider the heterogeneity of these patient populations and produce holistic models of disease.

In this study, we recruited a large multinational cohort of children across six LMICs to perform a comprehensive multiomic characterization of acute illness and identify biological pathways that mediate risk in this population. We employ proteomic, metabolomic, lipidomic, metagenomic, and pathogen profiling and demonstrate a signature of inpatient mortality that is persistent in the post-discharge period. By leveraging harmonized clinical data and a contrastive approach, we reveal patient subgroups with increased risk of death and the biological features that define them. Additionally, we show how the underlying biological correlates of mortality are impacted by patient characteristics such as malnutrition and age, with implications for clinical management. Finally, we distill all predictive models into a clinically-feasible biomarker set and validate our findings in an independent cohort of children, demonstrating the translational feasibility and generalizability of the study. Our results clarify the relationship between the biological mechanisms of acute illness and mortality and shed light on potential interventions to reduce child mortality in LMICs.

## Results

### Participants and study design

**Fig. 1:**
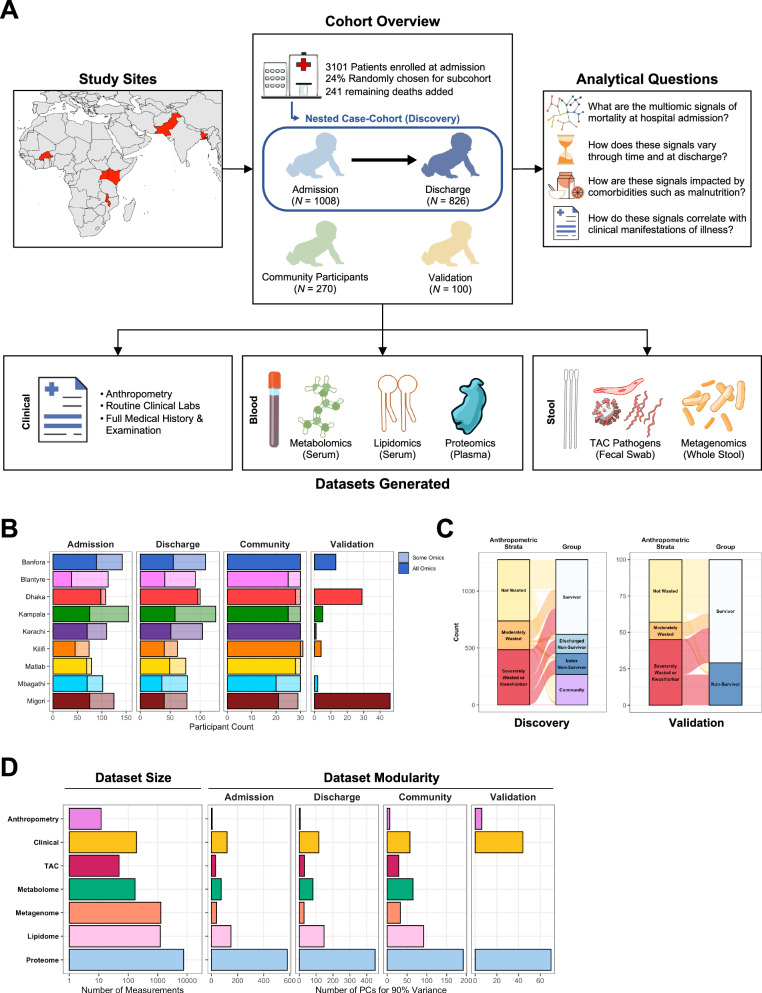
Study overview. **A** A cohort of 3101 children under 2 years of age were enrolled during hospital admission across 9 sites in 6 low- and middle-income countries (Kenya, Uganda, Malawi, and Burkina Faso in sub-Saharan Africa, and Bangladesh and Pakistan in south Asia) by the Childhood Acute Illness and Nutrition (CHAIN) Network study. Thirty geographically matched well community children per site were also selected from those enrolled. A nested case-cohort (NCC) cohort, hereafter referred to as the discovery cohort, was built with a random subcohort of 24% of the children plus all additional deaths, for a total of 1008 patients. Blood plasma and serum, fecal swabs, and whole stool taken at admission and discharge were used to generate lipidomics, metabolomics, proteomics, metagenomics, and TaqMan Array Card (TAC) pathogen datasets. Community participants were similarly profiled to generate matched multiomic datasets. A validation proteomic dataset was generated from a cohort of 100 children separate from the discovery using plasma samples taken at admission. **B** Distribution of participants by study site for the discovery cohort at admission and discharge, for community members, and for the validation cohort. Solid bars indicate the number of participants for which all omic datasets were generated, while shaded bars indicate additional patients for which only some omic datasets were generated. **C** Alluvial plot depicting the number of participants in each group stratified by their anthropometric strata for both the discovery and validation cohorts. **D** Characteristics of each of the datasets generated for the discovery cohort, for community members, and for the validation cohort. Left: Quantification of the number of measurements or features. Right: Estimation of the modularity—the degree of independent information between features in each dataset—using the number of principal components needed to explain 90% of the variance.

A cohort of 3101 hospitalized children was recruited by the Childhood Acute Illness and Nutrition (CHAIN) Network^28^ at hospital admission across nine sites in six LMICs (Fig. 1A). Clinical data, blood plasma and serum, fecal swabs, and whole stool were taken at admission and discharge. A nested case-cohort (NCC)^11^ was established within the study population to study mechanisms associated with mortality during hospitalization and in the 6-month post-discharge period (see “Methods” for details). The NCC, termed here as the discovery cohort, comprised 1008 participants, including 350 deaths (34.7%). A validation cohort of 100 children within the CHAIN study, independent of the discovery cohort, was profiled to assess the generalizability of the findings. Thirty geographically-matched, well community participants at each site (a total of 270 participants) were also included to determine norms within these communities. Clinical data included anthropometry, routine clinical laboratory assessments, medical history, and medical evaluation. Participant summary characteristics are listed in Tables 1 and 2, and a full list of the clinical variables measured can be found in the “Supplementary Materials”.

**Table 1:** Study participant characteristics

Characteristics	Discovery cohort (*N* = 1008)		Validation cohort (*N* = 100)		Community members (*N* = 270)	
	Number (percentage) or median [interquartile range]					
Outcome (died)	1008	350 (34.7%)	100	29 (29.0%)	269	-
Discharged	1008	826 (82.9%)	100	-	269	-
Age (months)	1008	10.6 [6.5, 15.8]	100	2.5 [1.0, 6.5]	269	12.2 [7.4, 17.5]
Weight (kg)	1005	6.4 [5.1, 7.8]	100	4.7 [3.4, 6.5]	269	8.3 [7.1, 9.3]
Height (cm)	1001	68.1 [62.5, 73.5]	100	57.2 [52.6, 68.9]	269	71.3 [65.6, 76.5]
Length-for-Age *z*-score	1001	−2.2 [−3.3, −1.2]	99	−1.7 [−2.9, −0.8]	269	−1.4 [−2.2, −0.8]
Weight-for-Age *z*-score	1005	−2.9 [−4.2, −1.6]	98	−2.1 [−4.1, −1.0]	269	−1.0 [−1.8, −0.2]
Weight-for-Length *z*-score	1000	−2.3 [−3.4, −1.1]	95	−1.3 [−2.4, −0.2]	269	−0.4 [−1.1, 0.4]
Sex (female)	1008	437 (43.3%)	100	42 (42.0%)	269	126 (46.8%)
Middle upper arm circumference (cm)	1008	11.8 [10.5, 12.8]	100	11.6 [9.5, 12.9]	269	13.6 [13.0, 14.5]
Head circumference (cm)	1005	42.9 [40.7, 44.9]	100	36.4 [35.3, 38.8]	269	44.1 [42.0, 46.0]
Oedema	1008	139 (13.8%)	100	1 (1.0%)	269	0 (0%)
HIV status	1008	76 (7.5%)	90	19 (21.1%)	269	3 (1.1%)
**Anthropometric strata**						
No wasting	1008	297 (29.5%)	100	43 (43.0%)	269	243 (90.3%)
Moderate wasting	1008	230 (22.8%)	100	12 (12.0%)	269	23 (8.6%)
Severe wasting or Kwashiorkor	1008	481 (47.7%)	100	45 (45.0%)	269	3 (1.1%)

**Table 2:** Number of participants per cohort stratified by site of origin or dataset

	Discovery cohort admission (*N* = 1008)	Discovery cohort discharge (*N* = 826)	Validation cohort (*N* = 100)	Community members (*N* = 270)
**Study Site**	**Number (Percentage)**			
Banfora	142 (14.1%)	109 (13.2%)	13 (13.0%)	30 (11.2%)
Blantyre	113 (11.2%)	92 (11.1%)	0 (0%)	30 (11.2%)
Dhaka	108 (10.7%)	100 (12.1%)	29 (29.0%)	30 (11.2%)
Kampala	155 (15.4%)	126 (15.3%)	5 (5.0%)	30 (11.2%)
Karachi	110 (10.9%)	104 (12.6%)	1 (1.0%)	30 (11.2%)
Kilifi	74 (7.3%)	62 (7.5%)	4 (4.0%)	30 (11.2%)
Matlab	79 (7.9%)	76 (9.2%)	0 (0%)	30 (11.2%)
Mbagathi	102 (10.1%)	79 (9.6%)	2 (2.0%)	30 (11.2%)
Migori	125 (12.4%)	78 (9.4%)	46 (46.0%)	29 (10.8%)
**Dataset**				
Anthropometry	1001 (99.3%)	825 (99.9%)	100 (100%)	269 (99.6%)
Clinical Variables	966 (95.8%)	802 (97.1%)	100 (100%)	265 (98.1%)
TAC	993 (98.5%)	739 (89.5%)	-	261 (96.7%)
Metabolome	924 (91.7%)	648 (78.5%)	-	262 (97.0%)
Metagenome	757 (75.1%)	575 (69.6%)	-	265 (98.1%)
Lipidome	906 (89.9%)	657 (79.5%)	-	260 (96.3%)
Proteome	945 (93.8%)	681 (82.4%)	100 (100%)	270 (100%)
Multiomic intersection	629 (62.4%)	466 (56.4%)	-	237 (87.8%)

The total number of discovery cohort participants at each site varied between 74 (Kilifi) and 155 (Kampala) (Fig. 1B). Participants in this cohort spanned anthropometric strata as determined by age, mid-upper arm circumference (MUAC), and oedema, with 297, 230, and 481 participants respectively classified as no wasting (NW, 29.5%), moderate wasting (MW, 22.8%), and severe wasting or kwashiorkor (SWK, 47.7%) (Fig. 1C). The distribution across anthropometric strata differed by outcome, with non-survivors showing a skew towards malnutrition (39 NW (11.15%), 62 MW (17.7%), and 249 SWK (71.15%)). Around half of the deaths in the discovery cohort occurred during the index hospitalization (182, 52.0%), with a median time to death of 3 days in this group. Conversely, participants who died during the post-discharge period had a median time to death of 55 days. The validation cohort included children across anthropometric strata (43 NW (43%), 12 MW (12%), and 45 SWK (45%)), and a total of 29 children in this cohort died (29%). The validation cohort did not include participants from Blantyre or Matlab (Fig. 1B).

### Multiomic profiling of the discovery cohort

Biological samples collected at hospital admission and discharge were analyzed to generate multiomic datasets, including proteomics from plasma, metabolomics and lipidomics from serum, metagenomics from whole stool, and TaqMan Array Card (TAC) pathogen detection from fecal swabs (Fig. 1A). Over half of all discovery participants had all omics successfully generated at admission (629, 62.4%) and discharge (466, 56.4%) (Fig. 1B). Most community participants had all omics successfully generated (237, 87.8%). The validation cohort underwent only proteomics profiling (100, 100%) due to cost and sample constraints and since proteomics provided the strongest signals differentiating survivors from non-survivors. A full breakdown of the number of participants per group with each omic profile can be found in Table 2.

Clinical data collected, which were harmonized through training across all sites, included a total of 202 measurements, of which 12 were anthropometric measures, and 190 were routine clinical and laboratory assessments (Fig. 1D). The targeted aptamer-based proteomics assay measured 6432 unique proteins using 7584 probes. The metabolomics and lipidomics profiling produced 170 and 1227 measurements, respectively. Finally, the metagenomic sequencing quantified the presence of 1276 microbes, and the TAC assay detected the presence of 36 different pathogens and 13 pathogenic genotypes, for a total of 10,306 multiomic features across all 5 omic modalities. Dataset modularity, or independent information content, was approximated with the number of principal components (PCs) needed to explain 90% of the variance in the data. Across all study groups, the proteomics data had the highest modularity, followed by the lipidome and the metabolome, with the metagenome and the TAC assay having the least amount of independent information (Fig. 1D).

### Multiomic modeling at admission predicts child mortality during hospitalization and post-discharge

**Fig. 2:**
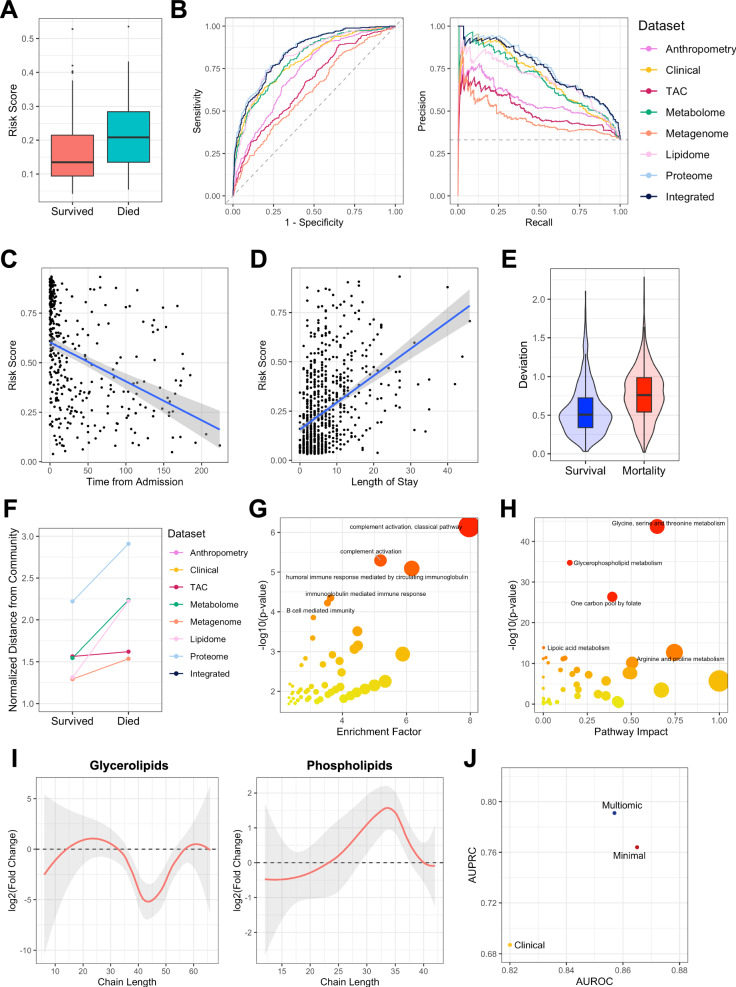
Multiomic modeling at admission predicts mortality during hospitalization and post-discharge. A cross-validated XGBoost model for the prediction of mortality during hospitalization or after discharge was trained on the integrated multiomic data of the discovery cohort. **A** Distribution of the mortality risk scores predicted by the multiomic model stratified by patient outcome (*N* = 1007 patients). **B** Cross-validated XGBoost models were built using each dataset separately for the prediction of mortality during hospitalization or after discharge and visualized with receiver-operating characteristic curves (left) and precision-recall curves (right). **C** Relationship between the multiomic model risk score and the time from admission to death for all cases. **D** Relationship between the multiomic model risk score and the time from admission to discharge for all discharged patients. **E** The distribution of feature deviation scores by time (see “Methods”) for each feature significantly associated with mortality or survival with at least a small effect size (|hedge’s *g* | > 0.2) (*N* = 2444 features). **F** Median normalized distance from the community stratified by outcome for each dataset analyzed (*N* = 629 patients). **G** Gene ontology (GO) overrepresentation analysis performed on the plasma proteome. Plot shows uncorrected two-sided p-values obtained using Fisher’s exact test. **H** Pathway analysis performed on the serum metabolome. Plot shows uncorrected one-sided p-values obtained using the Global test. **I** Lipid set enrichment analysis performed on glycerolipids and phospholipids quantified the association of lipid varieties with mortality or survival across lipid chain lengths. **J** Participants were randomly split into a training set (*N* = 807 patients, 80%) and a test set (*N* = 201 patients, 20%) to build a minimal XGBoost model for the prediction of mortality. Plot depicts the performance of the minimal, multiomic, and clinical models on the test set. Box plots indicate median (middle line); 25th and 75th percentiles (box limits); 1.5 × interquartile range (error bars); and outliers (single points). The colored lines and shadows in (**C,** **D**, **H**) represent the regression lines and the 95% Confidence Intervals, respectively. AUROC Area under the receiver operating characteristic curve. AUPRC Area under the precision recall curve.

The primary objective of the discovery cohort was to detect and quantify multiomic signals predictive of inpatient and post-discharge mortality in a heterogeneous population of acutely ill children. A cross-validated gradient-boosted tree model (XGBoost)^29^ built using the integrated multiomic data showed strong performance in the classification of mortality during hospitalization or in the post-discharge period (Wilcoxon *P* = 6.7 × 10^−72^, *N* = 1007) (Fig. 2A). The model architecture combined a repeated cross-validation scheme and an omic stacking procedure^30^, which enabled the generation of integrated multiomic predictions for every participant with at least one measured omic dataset (“Methods”). The multiomic model exhibited robust performance as assessed by the area under the receiver operating characteristic (ROC) curve (AUROC) of 0.84 (95% Confidence Interval (CI): 0.82–0.87) and the area under the precision-recall (PR) curve (AUPRC) of 0.75 (95% CI: 0.71–0.79) (Fig. 2B). The main features driving the predictive power of the model were generally related to immune, metabolic, and developmental processes, and included c-type lectin domain family 4 member C (CLEC4C) and fatty acid binding protein 3 (FABP3), SLIT and NTRK like family member 1 (SLITRK1), fatty acid binding protein 3 (FABP3), and SPARC related modular calcium binding 1 (SMOC1) (Table S1). Assay-specific models had varied performance in predicting mortality, with the proteome (*P* = 1.8 × 10^−69^, AUROC = 0.85) matching the predictive power of the multiomic model, followed by the lipidome (*P* = 3.8 × 10^−53^, AUROC = 0.82) and the metabolome (*P* = 6.0 × 10^−51^, AUROC = 0.80) (Fig. 2B and Table S2). In contrast, the fecal TAC and metagenomic models demonstrated AUROCs of 0.66 and 0.62, suggesting these modalities are weakly predictive of survival when measured at hospital admission. Notably, the multiomic model performed better than models built on either anthropometric or clinical data (AUROCs of 0.73 and 0.80, respectively).

The multiomic model achieved statistically significant performance across all nine study sites, with site-specific AUROCs ranging from 0.78 (Mbagathi) to 0.93 (Kilifi), (Fig. S1 and Table S3). Rural study sites (Banfora, Kilifi, Matlab, and Migori) showed higher model performance than urban study sites (Blantyre, Dhaka, Kampala, Karachi, and Mbagathi), with AUROCs and AUPRCs, respectively, of 0.88 and 0.81 for the rural participants compared to 0.81 and 0.69 for the urban participants. To assess the generalizability of the multiomic signature of mortality and quantify the confounding by site-specific factors, site-specific models were trained and evaluated across all other study sites (Fig. S2A). Interestingly, model performance depended more on the test site than the training site, with models from rural and urban areas performing similarly on the test site regardless of the test site’s characteristics (Fig. S2B). Importantly, training using all available sites led to the best performance overall, underscoring the importance of heterogeneity during training for maximizing generalizability. These findings were supported by the evaluation of site-confounding on the univariate association between multiomic features and mortality. The non-adjusted coefficient estimates of association between each feature and mortality on site-normalized data were highly correlated with the estimates derived from random intercept (Spearman’s *rho* (*rho*) = 0.97, *P* = 0) (Fig. S2C), random intercept and random slopes (*rho* = 0.94, *P* = 0) (Fig. S2D), and meta-regression models (*rho* = 0.95, *P* = 0) (Fig. S2E). Overall, this analysis demonstrated that the multiomic signals of mortality are broadly site-independent and that training across heterogeneous sites leads to improved overall model performance.

Another important aspect to evaluate the multiomic model of mortality is its performance across time, especially in clarifying the extent to which these signals are primarily caused by disease severity at admission versus underlying biological risk. Multiomic mortality risk scores were negatively correlated with the time to death for cases (*rho* = −0.52, *P* = 3.8 × 10^−25^) (Fig. 2C) and positively correlated with the length of hospital stay among discharged participants (*rho* = 0.46, *P* = 1.2 × 10^−41^) (Fig. 2D). Furthermore, the performance of the model decreased when trying to predict deaths which occurred farther from the time of hospital admission (Fig. S3A–D). To better quantify the impact of time from admission on the multiomic signature of mortality, cases were binned into three time intervals (<3 days, 3–14 days, and >14 days). “Deviation scores”, a measure of how a feature’s deleterious or protective effect varied across these time intervals, were then calculated for all features significantly associated with either survival or mortality with at least a small effect size (|hedge’s *g* | > 0.2) (“Methods”). Interestingly, features associated with mortality exhibited significantly higher deviation scores than those associated with survival (Wilcoxon *P* = 3.7 × 10^−54^) (Figs. 2E and S3E, F). Models trained using only the cases within one specific time interval and tested in the other two achieved generally consistent performance (Fig. S3G) and moderate correlation between model predictions (Fig. S3H). The greatest disagreement, while small, was seen between the models trained with the early (<3 days) and late (>14 days) deaths, possibly due to the earlier group including children with irreversible mortality-associated changes. Altogether, these results indicate that multiomic profiling captured multiple, potentially non-overlapping biological risks, with higher predictive power for short-term mortality.

Mortality risk is influenced by a variety of factors, such as biology, environment, and socioeconomic status. Thus, the relationship between these factors and the multiomic signature of mortality was investigated. As part of the primary CHAIN cohort analysis of mortality, latent factors were generated as summary scores of the variables measured across these domains. All but one of these latent factors were significantly correlated with the multiomic mortality risk scores, with correlations ranging from weak to moderate (Fig. S4). Similarly, the clinical signs of disease present at admission reflect factors that drive mortality risk, so their association with the multiomic signature of mortality was also assessed. The main clinical signs significantly associated with the multiomic mortality risk score were reflective of malnutrition, septic shock, or HIV-related conditions; however, common infectious diseases like pneumonia and diarrhea were not significantly associated with the multiomic signature (Fig. S5). Overall, the multiomic signature of mortality captured many of the factors influencing mortality risk without being dominated by any specific one.

To gain a systems-level view of the impact of acute illness on the different biological systems profiled, two different approaches were used. First, by computing the normalized omic distance of each participant in relation to the reference omic landscape provided by the community members (“Methods”). These distances measured how generally perturbed a specific omic was in a participant in relation to the median community member across all sites. The lipidome (Wilcoxon *P* = 1.6 × 10^−16^), metabolome (Wilcoxon *P* = 9.4 × 10^−15^), and proteome (Wilcoxon *P* = 1.6 × 10^−7^) showed significantly higher normalized omic distances in cases versus controls, and all omics were significantly perturbed in the discovery cohort compared to the community participants (Fig. 2F).

Second, by using protein, metabolite, and lipid pathway analyses. Protein set overrepresentation analysis for Gene Ontology (GO) terms^31–33^ performed on the plasma proteomic features significantly associated with mortality or survival with at least a medium effect size (|hedge’s *g* | > 0.5) revealed an enrichment in multiple immunity-related GO terms, such as complement activation (Fisher’s exact test (Fisher’s) *P* = 5.1 × 10^−6^) and immunoglobulin mediated immune response (Fisher’s *P* = 4.5 × 10^−5^) (Fig. 2G). Metabolite pathway analysis^34^ performed on the 123 measured metabolites annotated in the Kyoto Encyclopedia of Genes and Genomes (KEGG) database revealed significant alterations to multiple metabolic pathways, including glycine, serine and threonine metabolism (*P* = 1.0 × 10^−42^) and arginine and proline metabolism (*P* = 1.8 × 10^−12^) (Fig. 2H). Notably, the metabolomic signature of mortality validated the findings from a previous study performed on a similar pediatric cohort (*rho* = 0.73, *P* = 2.3 × 10^−14^) (Fig. S6)^10^. Lipid set enrichment analysis^35,36^ performed on the lipids significantly associated with mortality or survival indicated the enrichment of glycerolipids and phospholipids of specific chain lengths (Fig. 2I).

Distilling multiomic predictive models to a small number of features is critical to maximize their translational potential in LMICs. To examine the feasibility of a minimal model for the prediction of mortality in these settings, participants were randomly split into a training set (*N* = 807, 80%) and a test set (*N* = 201, 20%). A feature selection procedure (“Methods”) was used in the training set to select 10 multiomic features (9 proteins and 1 lipid, Table S4). Using these 10 features and all 12 anthropometric features, a minimal XGBoost model was trained on the training set and applied on the test set. The minimal model achieved the performance of the full multiomic model (Wilcoxon *P* = 1.7 × 10^−17^, *N* = 201), with an AUROC of 0.87 (95% CI: 0.81–0.92) and an AUPRC of 0.76 (95% CI: 0.68–0.85) (Figs. 2J and S7). The strong performance of the minimal model demonstrates the translational feasibility of the predictive model for mortality.

### Contrastive analysis of the multiomic and clinical models of mortality reveals patient subgroups

**Fig. 3:**
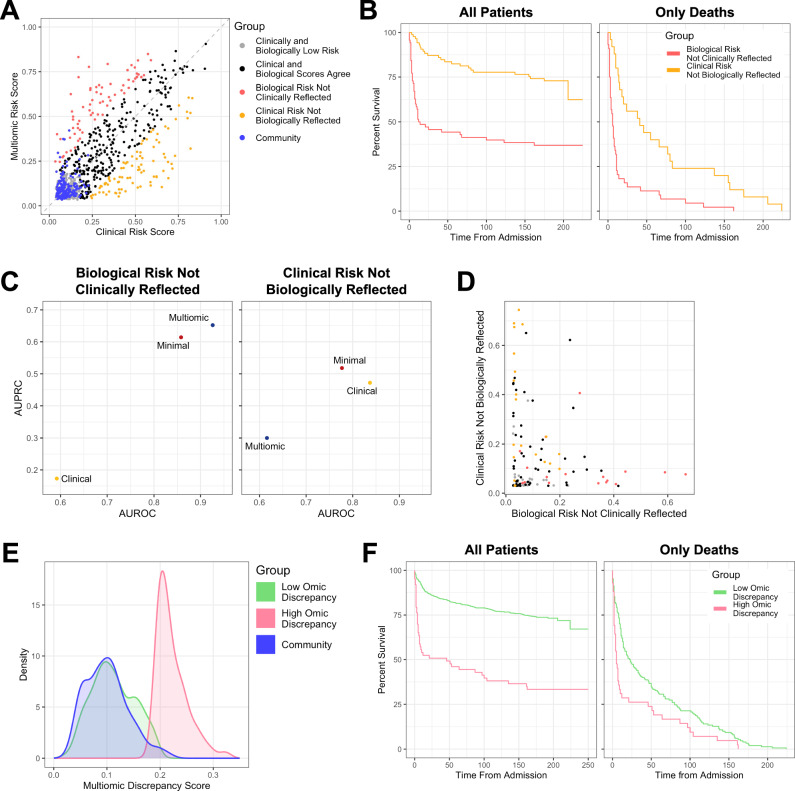
Contrastive analysis of the multiomic and clinical models of mortality reveals patient subgroups with different survival patterns. **A–C** Cross-validated XGBoost models for the prediction of mortality during hospitalization or after discharge were trained on the clinical data and the integrated multiomic data for discovery cohort members with all omics datasets available (*N* = 629 patients). The risk scores of community members based on these models were then calculated. **A** Comparison between the mortality risk scores predicted by the multiomic and the clinical models, with colors representing patient subgroups. **B** Kaplan–Meier survival curves for the discrepant subgroups defined in (**A**), showing the survival curves based on all patients (left) or only on patients that died (right). **C**, **D** Participants were randomly split into a training set (*N* = 504 patients, 80%) and a test set (*N* = 125 patients, 20%) to build a minimal XGBoost model for the classification of patients into discrepant subgroups. **C** Plots depict the performance of the minimal, multiomic, and clinical models on the test set. **D** Comparison between the risk scores predicted by the minimal model. **E**, **F** XGBoost models for the prediction of mortality during hospitalization or after discharge were trained on each omic dataset for the subset of discovery cohort members with all omics datasets available (*N* = 629 patients). The risk scores of community members based on these models were then calculated. **E** Distribution of multiomic discrepancy scores, with colors representing patient subgroups. **F** Kaplan–Meier survival curves for the discrepant subgroups defined in (**E**), showing the survival curves based on all patients (left) or only on patients that died (right).

Children hospitalized with severe illness may unexpectedly deteriorate without preexisting severe signs or respond rapidly to clinical care^37,38^. Thus, this study also aimed to define how the multiomic and clinical signals of mortality align and contrast by comparing models built on either multiomic data or anthropometric measures, routine clinical assessments, and laboratory tests. The difference between the risk scores predicted by these two models was significantly higher in the cases than in the controls (Wilcoxon *P* = 2.9 × 10^−18^, *N* = 965) (Fig. S8A). To better understand the biology of participants with discrepant multiomic and clinical phenotypes, participants were then classified into groups based on the concordance between their respective model risk scores (Fig. 3A). For this analysis, only the 629 participants with complete multiomic profiling were used in order to ensure equal contributions from all biological modalities. If both scores indicated similarly low or high levels of risk, participants were respectively included in the Clinically and Biologically Low Risk (*N* = 195) or Clinical and Biological Scores Agree (*N* = 277) groups (“Methods”). Otherwise, participants were included in the Biological Risk not Clinically Reflected (*N* = 70) or the Clinical Risk not Biologically Reflected (*N* = 87) groups, depending on which risk score was higher.

Strikingly, the risk of death in the Biological Risk not Clinically Reflected group was significantly higher than in the Clinical Risk not Biologically Reflected group (Cox Proportional-Hazard (Cox) *P* = 1.2 × 10^−6^), suggesting that biological risk, as determined here, is more reliable than the clinical assessments conducted (Fig. 3B). Participants in the former group also died significantly faster than those in the latter (Cox *P* = 3.7 × 10^−5^) (Fig. 3B), but participants in both groups were discharged at similar rates (Cox *P* = 0.42) (Fig. S8B). Univariate analysis of the features most different between these two groups revealed that participants in the Clinical Risk not Biologically Reflected group consisted of children with severe malnutrition with reduced expression of the markers of inflammation and immune activity, which characterized the children in the Biological Risk not Clinically Reflected group (Fig. S9). GO term overrepresentation analysis of the proteins most different between these two groups indicated enrichment of the antimicrobial humoral response (Fisher’s *P* = 9.1 × 10^−5^) and, interestingly, limb development (Fisher’s *P* = 2.5 × 10^−5^) (Fig. S8C). The mid-upper arm circumference (MUAC) of the Clinical Risk not Biologically Reflected group was especially low, even when compared against the Clinical and Biological Scores Agree group (Wilcoxon *P* = 1.3 × 10^−10^) (Fig. S8D).

Given the clinical relevance of the discrepant patient subgroups found, minimal models were developed as previously described to classify participants into either the Biological Risk not Clinically Reflected or the Clinical Risk not Biologically Reflected groups. For this task, participants were again randomly split into a training set (*N* = 504, 80%) and a test set (*N* = 125, 20%), with the same feature selection paradigm used to select 10 multiomic features to build the minimal models (Table S5). Two minimal models - one for the classification of each discrepant subgroup - were trained on the training set with the minimal features and all 12 anthropometric features and applied to the test set. Both models demonstrated robust performance (Fig. 3C); in particular, the minimal model could detect patients in the Biological Risk not Clinically Reflected (Wilcoxon *P* = 9.5 × 10^−7^, AUROC = 0.87, AUPRC = 0.63, *N* = 125), which the clinical model failed to classify correctly (Wilcoxon *P* = 0.47, AUROC = 0.56, AUPRC = 0.16, *N* = 125). As expected, minimal model predictions for each discrepant subgroup were mutually exclusive (Fig. 3D). The strong performance of the minimal models in identifying these clinically-relevant patient subgroups paves the way for targeted intervention based on improved risk identification and better understanding of the impact of malnutrition on clinical features.

Acute illness can also manifest differently across biological systems, causing children to exhibit more complex biological profiles that can hinder therapeutic intervention based on simple clinical syndromes^11^. To investigate this phenomena, multiomic discrepancy scores, defined as the standard deviation of a participant’s omic risk scores, were used to quantify the disagreement of mortality risk across biological systems (“Methods”). The multiomic discrepancy scores were significantly higher in the cases than in the controls (Wilcoxon *P* = 3.8 × 10^−11^) (Fig. S10A). To investigate the key features which characterized high multiomic discrepancy, the participants with multiomic discrepancy scores above the 90th percentile were included in the High Omic Discrepancy (*N* = 63) group, with the rest of the participants in the Low Omic Discrepancy (*N* = 566) group (Fig. 3E). Participants with high multiomic discrepancy were significantly more likely to die (Cox *P* = 1.6 × 10^−11^) and died more quickly (Cox *P* = 0.005) than those with lower multiomic discrepancy (Fig. 3F). However, there was no difference in how quickly these patients were discharged from the hospital (Cox *P* = 0.72) (Fig. S10B).

Remarkably, univariate analysis of the features associated with High Omic Discrepancy revealed a clinical picture compatible with septic shock, in alignment with previous studies in other cohorts^23^ (Fig. S11). GO term overrepresentation analysis of the proteins associated with high omic discrepancy indicated enrichment of the defense response to fungus (Fisher’s *P* = 1.3 × 10^−4^) and multiple RNA processing terms such as regulation of mRNA processing (Fisher’s *P* = 4.0 × 10^−5^), potentially a response to viral infection in these patients^39,40^ (Fig. S10C). The clinical condition of the High Omic Discrepancy group at hospital admission was characterized by elevated creatinine levels (Benjamini–Hochberg (BH)-adjusted Wilcoxon *P* = 9.2 × 10^−4^), reduced levels of albumin (BH-adjusted Wilcoxon *P* = 0.002), and symptoms such as poor capillary refill (BH-adjusted Wilcoxon *P* = 9.4 × 10^−5^), cold peripheries (BH-adjusted Wilcoxon *P* = 7.1 × 10^−5^), and a lower consciousness level (BH-adjusted Wilcoxon *P* = 0.002) (Fig. S10D, E). Overall, understanding the underlying biology and clinical presentations of these patient subgroups at increased risk of death paves the way for the development of targeted therapeutic interventions to mitigate this risk.

### Multiomic modeling of mortality in the post-discharge period indicates a persistent disease signature in discharged patients

**Fig. 4:**
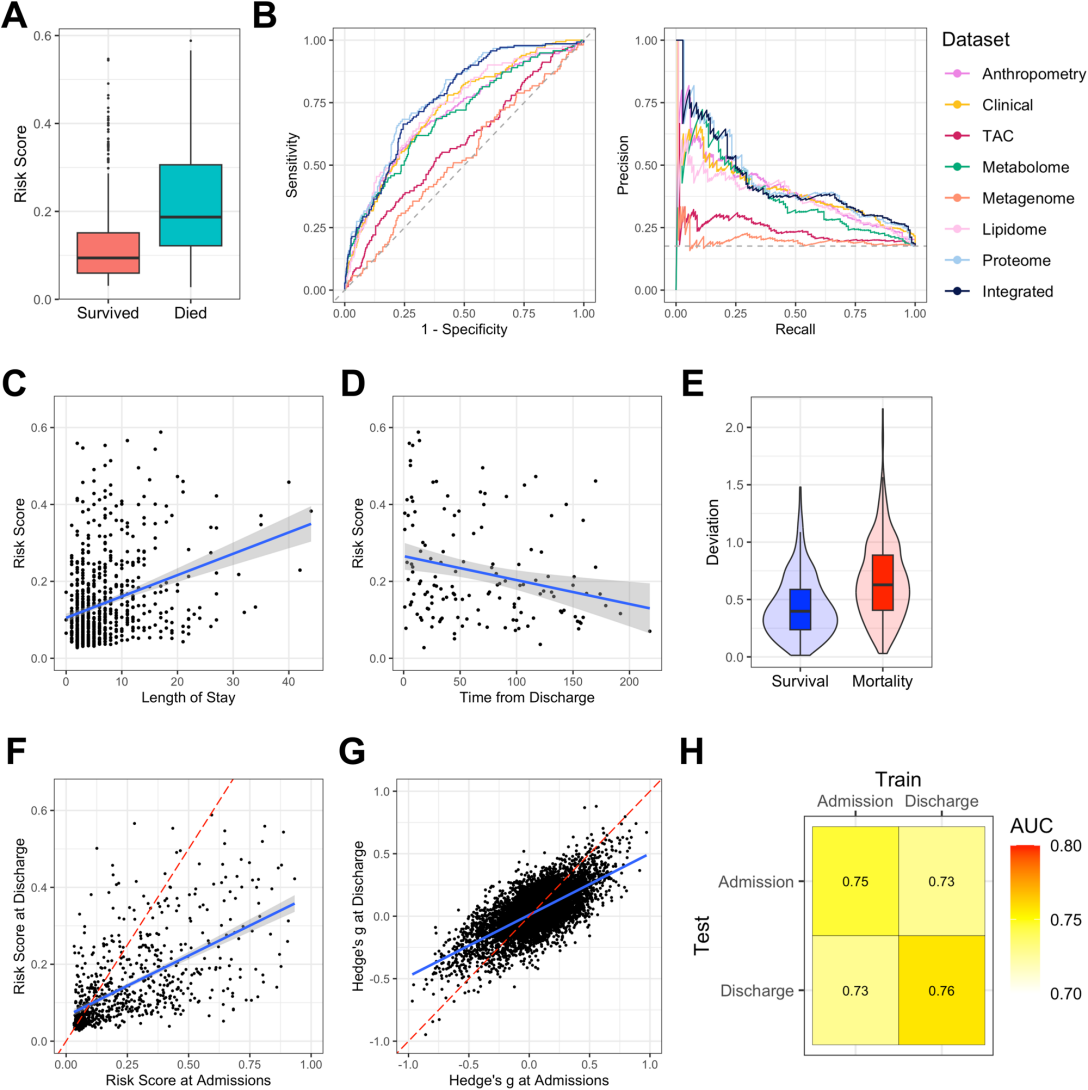
Multiomic modeling of mortality in the post-discharge period indicates a persistent disease signature in discharged patients. A cross-validated XGBoost model for the prediction of mortality after discharge was trained on the integrated multiomic data of the discovery cohort. **A** Distribution of the mortality risk scores predicted by the multiomic model stratified by patient outcome (*N* = 750 patients). **B** Cross-validated XGBoost models were built using each dataset separately for the prediction of mortality after discharge and visualized with receiver-operating characteristic curves (left) and precision-recall curves (right). **C** Comparison of the multiomic model risk score and the time from admission to death for all cases. **D** Comparison of the multiomic model risk score and the time from admission to discharge for all discharged patients. **E** The distribution of feature deviation scores by time for each feature significantly associated with mortality or survival with at least a small effect size (|hedge’s *g* | > 0.2) (*N* = 676 features). **F** Comparison of the multiomic model risk scores for the prediction of mortality using data collected at hospital admission or discharge. **G** Comparison of the hedge’s *g* for each feature’s association with mortality or survival at hospital admission and discharge. **H** Cross-validated XGBoost models for the prediction of mortality after discharge were trained on the integrated multiomic data of discovery cohort patients with data at both hospital admission and discharge (*N* = 826 patients). Models were then used to predict mortality using data from the other timepoint and their performance evaluated using the AUROC. Box plots indicate median (middle line); 25th and 75th percentiles (box limits); 1.5 × interquartile range (error bars); and outliers (single points). The blue lines and shadows in (**C**, **D**, **F**, **G**) represent the regression lines and 95% Confidence Intervals, respectively. AUC Area under the curve.

Children may not be fully recovered at hospital discharge, as shown in the discrepancy groups having the same rates of discharge, leading to risks of readmission and post-discharge mortality. This study also sought to characterize the multiomic signals of mortality present at discharge and determine how these compared to those at admission. An XGBoost model built using the integrated multiomic data collected at discharge showed moderate performance in predicting mortality in the post-discharge period (Wilcoxon *P* = 1.5 × 10^−22^, *N* = 750), with an AUROC of 0.77 (95% CI: 0.72–0.81) and an AUPRC of 0.44 (95% CI: 0.37–0.51) (Fig. 4A, B). Similarly to hospital admission, the proteome (*P* = 1.9 × 10^−21^, AUROC = 0.78), the lipidome (*P* = 3.6 × 10^−15^, AUROC = 0.73) and the metabolome (*P* = 3.2 × 10^−11^, AUROC = 0.70) had the best performance among the different omics profiled (Fig. 4B and Table S6). Unlike the admissions model, the discharge model showed better performance in the urban study sites than the rural ones, with AUROCs of 0.76 and AUPRCs of 0.49 and 0.36 for urban and rural sites, respectively, potentially because of higher wealth and access to care in the urban sites. The discharge model also failed to achieve significant performance across all study sites, with lower performance at the Banfora, Kampala, and Migori sites (Fig. S12 and Table S7). These results could be explained by different patterns of hospital use between populations and by heterogeneity in discharge guidelines across sites.

The mortality risk scores predicted by the multiomic model were positively correlated with the length of hospital stay preceding discharge (*rho* = 0.32, *P* = 3.9 × 10^−19^) (Fig. 4C) and negatively correlated with the time to post-discharge death (*rho* = −0.26, *P* = 0.002) (Fig. 4D). Similar to the model at hospital admission, model performance decreased with time (Fig. S13A–D), and examination of the feature deviation scores at discharge also showed significantly higher deviation scores for the mortality-associated features than those associated with survival (Wilcoxon *P* = 4.0 × 10^−20^) (Figs. 4E and S13E, F). Unlike at hospital admission, only the metabolome (Wilcoxon *P* = 1.5 × 10^−6^) and the lipidome (Wilcoxon *P* = 0.005) showed significantly higher normalized omic distances in non-survivors (Fig. S14). The multiomic discrepancy scores at discharge were also significantly higher in cases than in controls (Wilcoxon *P* = 3.7 × 10^−4^) (Fig. S15A), with an increased risk of mortality in patients in the High Omic Discrepancy group (Cox *P* = 7.1 × 10^−4^) (Fig. S15B, C). Patients in this group did not die at faster rates than the Low Omic Discrepancy group (Cox *P* = 0.24), but they did exhibit a slightly longer length of hospital stay preceding discharge (Cox *P* = 0.009) (Fig. S15D, E). The patients with discrepant clinical and multiomic phenotypes at discharge had statistically indistinguishable mortality characteristics (Fig. S16).

All children in this study received guideline-directed care after admission, so that all deaths, and especially post-discharge ones, occurred despite treatment. Thus, to clarify the mechanisms underlying post-discharge mortality, it is critical to understand the impact of hospitalization on the biological signatures of mortality. Two approaches were used to examine this impact, starting with a direct comparison of the multiomic signatures of mortality at admission and discharge. Despite the differences found in the two multiomic models, the overarching biological signals displayed a high degree of similarity. The multiomic risk scores output by each model for the same participants were highly correlated (Pearson’s *r* (*r*) = 0.60, *P* = 1.3 × 10^−73^) (Figs. 4F and S17), as were the effect sizes of the associations of each feature to mortality at admission and discharge (*r* = 0.67, *P* = 0) (Fig. 4G). Moreover, multiomic models trained on either hospital admission or discharge and tested with the corresponding data at the other timepoint showed very similar performance, indicating the multivariate signals of mortality are shared between admission and discharge (Fig. 4H).

Second, the change in multiomic profiles for patients with data at both admission and discharge was assessed for its association with post-discharge mortality. An XGBoost model built using these changes showed weak performance in predicting mortality in the post-discharge period (Wilcoxon *P* = 3.8 × 10^−11^, *N* = 748), with an AUROC of 0.68 (95% CI: 0.63–0.73) and an AUPRC of 0.32 (95% CI: 0.27–0.39) (Fig. S18A, B). All omics showed a reduction in normalized omic distances between hospital admission and discharge, with the highest reduction in the metagenome (Wilcoxon *P* = 5.3 × 10^−8^) (Fig. S18C). Accordingly, 4721 (45.8%) multiomic features showed significant changes between admission and discharge. However, only 92 (0.9%) features had changes that were significantly associated with mortality (Fig. S18D). Altogether, these results indicate that the biological causes of disease and mortality in the post-discharge period largely mirror the signals observed at hospital admission despite hospitalization and treatment.

### Anthropometric strata, but not age, impact the multiomic signature of mortality

**Fig. 5:**
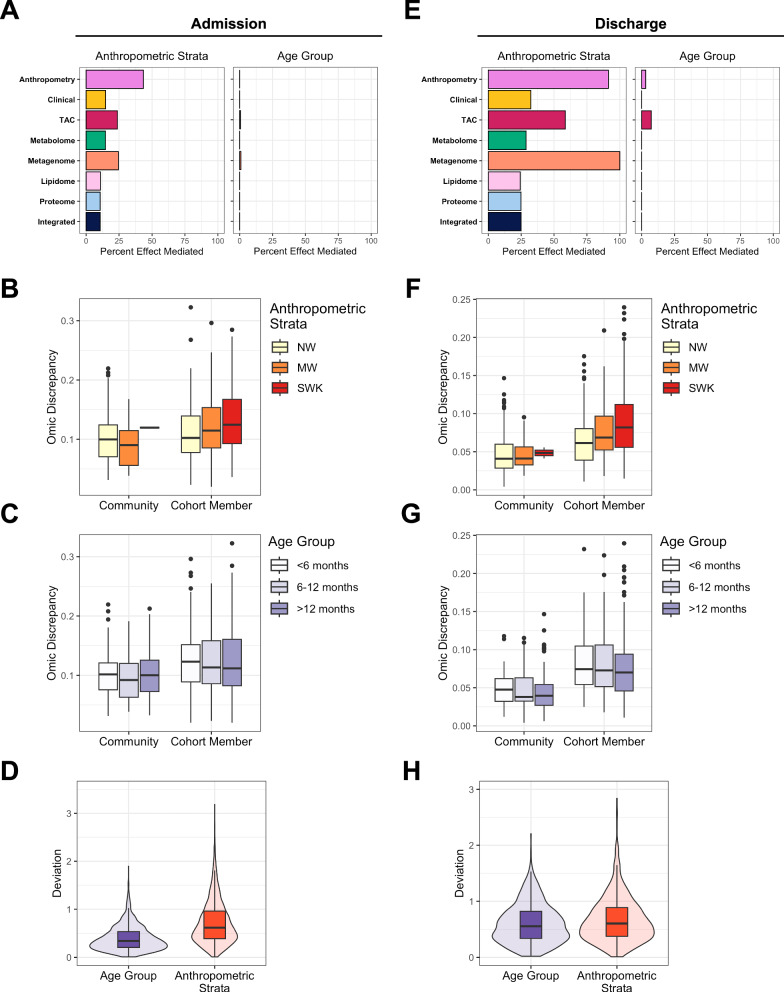
Anthropometric strata, but not age, impact the multiomic signature of mortality. Cross-validated XGBoost models were built using each dataset separately for the prediction of mortality during hospitalization or after discharge using data collected at hospital admission (**A–D**) or for the prediction of mortality after discharge using data collected at discharge (**E–H**). **A** Mediation analysis of the mortality risk scores at hospital admission using either middle upper arm circumference (MUAC, left) or age (right) as a mediator. Multiomic discrepancy scores at hospital admission stratified by anthropometric strata (**B**) or age group (**C**) (*N* = 639 patients). **D** The distribution of feature deviation scores at hospital admission by anthropometric strata and age group for each feature significantly associated with mortality or survival with at least a small effect size (|hedge’s *g* | > 0.2) (*N* = 2443 features). **E** Mediation analysis of the mortality risk scores at discharge using either MUAC (left) or age (right) as a mediator. Multiomic discrepancy scores at discharge stratified by anthropometric strata (**F**) or age group (**G**) (*N* = 466 patients). **H** The distribution of feature deviation scores at discharge by anthropometric strata and age group for each feature significantly associated with mortality or survival with at least a small effect size (|hedge’s *g* | > 0.2) (*N* = 675 features). Box plots indicate median (middle line); 25th and 75th percentiles (box limits); 1.5 × interquartile range (error bars); and outliers (single points). NW Not wasted, MW Moderately wasted, and SWK Severely wasted or Kwashiorkor.

Certain patient characteristics and/or comorbidities can increase the complexity of disease presentation and complicate effective clinical care. In particular, sick children at LMICs who are younger or undernourished can be particularly hard to treat^16,41^. The discovery cohort presented an unprecedented opportunity to investigate the impact of these characteristics on the multiomic signals of mortality. Mediation analysis of the mortality risk scores based on hospital admission showed that the MUAC, but not age, mediated the relationship between the model predictions and mortality (Fig. 5A). Accordingly, the performance of the multiomic model dropped in the MW and SWK groups, whereas model performance was more consistent across age groups (Fig. S19A).

Malnutrition also showed an increased impact than age on systems-level biological metrics. Multiomic discrepancy scores were significantly higher in the SWK (Wilcoxon *P* = 1.4 × 10^−5^) and MW (Wilcoxon *P* = 0.04) groups when compared to the NW group, whereas multiomic discrepancy scores were statistically indistinguishable between age groups (Fig. 5B, C). The normalized omic distances of participants in the SWK group were significantly higher than the NW group for all omics except the Metagenome, whereas age groups showed less significant differences between groups (Fig. S19B and Table S8). Feature deviation scores were significantly higher within anthropometric strata than in age groups (Wilcoxon *P* = 2.3 × 10^−171^) (Fig. 5D), driven primarily by an increase in feature deviation scores in mortality-associated features (Fig. S20A). Interestingly, there was no difference in feature deviation scores between mortality- and survival-associated features within age groups (Wilcoxon *P* = 0.77), whereas mortality-associated features showed higher feature deviation scores than survival-associated ones when within anthropometric strata (Wilcoxon *P* = 1.2 × 10^−28^) (Fig. S20B), mirroring the results seen when stratifying patients by time (Fig. 2E).

The interactions between undernutrition, age, and the multiomic signals of mortality at discharge closely paralleled the results found at hospital admission. Patient MUAC but not age showed significant mediation of the mortality risk scores and patient outcomes (Fig. 5E). Unlike the admission models, models for the prediction of post-discharge mortality showed reduced performance both across anthropometric strata and by age groups (Fig. S21A). As in hospital admission, multiomic discrepancy scores were significantly higher in the SWK (Wilcoxon *P* = 2.0 × 10^−8^) and MW (Wilcoxon *P* = 0.01) groups when compared to the NW group, with no difference between age groups (Fig. 5F, G). The proteome, lipidome, and metabolome all showed significantly higher normalized omic distances in the SWK, with the metagenome showing the only significant difference between two age groups (Fig. S21B and Table S9). In contrast to hospital admission, feature deviation scores were only slightly higher within anthropometric strata than in age groups (Wilcoxon *P* = 2.9 × 10^−4^) (Fig. 5H), driven solely by an increase in the survival-associated features (Fig. S22A). Surprisingly, there was a small difference between mortality- and survival-associated features within anthropometric strata (Wilcoxon *P* = 0.007), while the scores within age groups showed a notable increase in mortality-associated feature deviation scores (Wilcoxon *P* = 7.2 × 10^−14^) (Fig. S22B). On balance, these results suggest that the biological processes associated with acute illness are substantially more impacted by the patient’s nutritional status than by age, both at hospital admission and discharge.

### Validation in an independent cohort demonstrates generalizability

**Fig. 6:**
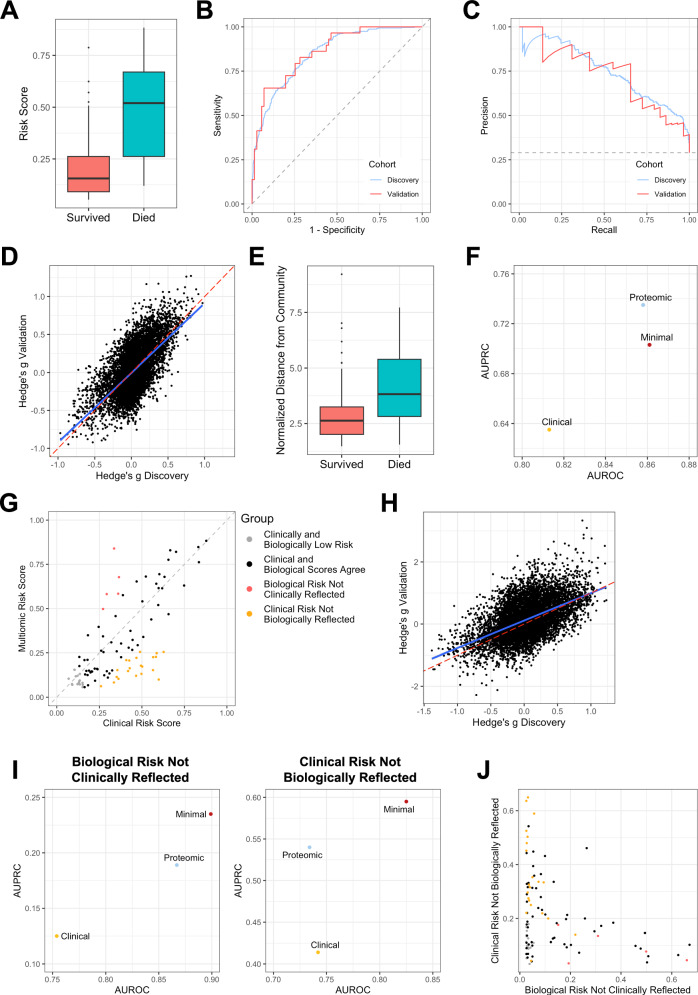
Validation in an independent cohort demonstrates generalizability. The cross-validated XGBoost models for the prediction of mortality trained on the proteomics data and the clinical data of the discovery cohort were applied on the validation cohort. **A** Distribution of the mortality risk scores predicted by the discovery proteomics model for the validation cohort stratified by patient outcome (*N* = 100 patients). **B** Receiver-operating characteristic curves for the cross-validated discovery proteomics model applied on the discovery cohort and the validation cohort. **C** Precision-recall curves for the cross-validated discovery proteomics model applied on the discovery cohort and the validation cohort. **D** Comparison of the hedge’s *g* for each feature’s association with mortality or survival at hospital admission for the discovery and validation cohorts. **E** Median normalized distance from the community stratified by outcome for the proteome in the validation cohort (*N* = 100 patients). **F** The minimal, proteomic, and clinical XGBoost models for the prediction of mortality trained on the discovery cohort were applied on the validation cohort. Plot depicts the performance of the minimal, proteomic, and clinical models on the validation cohort. **G** Comparison between the mortality risk scores predicted by the discovery proteomics model and discovery clinical model on the validation cohort, with colors representing patient subgroups. **H** Comparison of the hedge’s *g* for each feature’s association with the discrepant subgroups defined in (**G**) and Fig. 3A. **I, J** The minimal, proteomics, and clinical XGBoost model for the classification of patients into discrepant subgroups trained on the discovery cohort was applied to the validation cohort. **I** Plots depict the performance on the validation cohort of the minimal, proteomic, and clinical models. **J** Comparison between the risk scores on the validation cohort predicted by the minimal model. Box plots indicate median (middle line); 25th and 75th percentiles (box limits); 1.5 × interquartile range (error bars); and outliers (single points). The blue lines and shadows in (**D**, **H**) represent the regression lines and the 95% Confidence Intervals, respectively. AUROC Area under the receiver operating characteristic curve. AUPRC Area under the precision recall curve.

Independent validation of results found in multiomic studies is critical to demonstrate generalizability^42^. Thus, an additional 100 patients from the larger CHAIN cohort were profiled to generate a proteomics dataset at hospital admission to investigate whether the findings from the discovery cohort could be replicated more broadly. Strikingly, the proteomic model trained using the discovery data at hospital admission for the prediction of mortality during hospitalization or in the post-discharge period demonstrated consistent performance in the validation cohort (Wilcoxon *P* = 2.2 × 10^−8^, *N* = 100) (Fig. 6A). The discovery proteomics model applied on the validation data achieved an AUROC of 0.86 (95% CI: 0.78–0.94) and an AUPRC of 0.74 (95% CI: 0.59–0.87) (Fig. 6B, C), closely mirroring the cross-validated performance seen in the discovery cohort (AUROC = 85, AUPRC = 0.75). Univariate feature associations with mortality or survival between the two cohorts were also highly correlated (*r* = 0.65, *P* = 0) (Fig. 6D). As in the discovery cohort, the normalized distance from the community for the proteome was higher in cases than in controls (Wilcoxon *P* = 0.001) (Fig. 6E). A minimal model trained on the discovery cohort using 10 proteomic features (Table S10) and all 12 anthropometric features also achieved strong performance on the validation cohort (Wilcoxon *P* = 1.7 × 10^−8^, AUROC = 0.86, AUPRC = 0.70, *N* = 100), demonstrating both generalizability and translational feasibility (Figs. 6F and S23).

Furthermore, patient subgroups defined based on discrepant proteomic and clinical risk scores obtained by applying the discovery models to the validation cohort displayed a concordant phenotype to the respective subgroups in the discovery cohort (Fig. 6G). The percentage of patients that died within each subgroup in the validation cohort paralleled the discovery cohort (*r* = 0.95, *P* = 0.05) (Fig. S24), as were the features that characterized the discrepant patient subgroups (*r* = 0.53, *P* = 0) (Figs. 6H and S25). The minimal models for the classification of patients into discrepant groups trained on the discovery cohort also generalized to the validation cohort, underscoring the potential of this approach for improving risk stratification in LMICs (Fig. 6I, J and Table S11).

In summary, these results provide an unprecedented comprehensive multiomic characterization of mortality in sick children across LMICs, with key implications for clinical management and therapeutic interventions in these settings.

## Discussion

In this study, we leveraged the CHAIN Network’s large and geographically diverse cohort with harmonized clinical data and biological sample collection to examine the complex biological mechanisms of childhood acute illness and mortality in LMICs. Using simultaneous high-dimensional proteomic, metabolomic, lipidomic, metagenomic, and pathogen profiling, we uncovered a persistent multiomic disease signature that contributes to acute illness and mortality. Our cohort design also enabled the interrogation of how this signature varies along anthropometric strata, age groups, and through time. With the rich clinical data collected at hospital admission, we defined patient subgroups based on their distinct manifestations of disease and how these manifestations aligned with their biology. To establish the translational feasibility of our approach and maximize the potential impact of our study, we distilled the predictive models into a clinically relevant set of biomarkers with a minimal loss in performance. Finally, we validated our findings in an independent cohort of acutely ill children, demonstrating the robustness and generalizability of the study results.

The integrated multiomic model of mortality during hospitalization or in the post-discharge period achieved an AUROC of 0.84, in line with previous studies showing the potential of biological profiling in predicting outcomes in critically ill patients^43–46^. Our work extends this to a large, heterogeneous pediatric population in LMICs, while also showing that these multiomic signals outperform traditional clinical and anthropometric measures, with implications for risk stratification. Interestingly, the model’s performance varied across study sites and between rural and urban settings, which could be attributed to local variations in environmental exposures and healthcare practices^22,42,47^. However, the multiomic signature of mortality generalized better when inferred using heterogeneous sites, so that site-specific performance probably reflected distributional differences rather than mechanistic ones. This interpretation is further supported by the limited association between the different domains of exposures and the multiomic signature of mortality. Thus, while the multiomic signature might be capturing the effect of these factors on mortality risk, it is still mostly driven by generalizable biology.

Features associated with mortality included systemic perturbations in immune, inflammatory, and metabolic processes. Consequently, the proteome, lipidome, and metabolome were also the most individually predictive of mortality. Similarly, many clinical signs and symptoms at admission were also significantly associated with the biological signatures found, with the strongest associations found for signs of malnutrition, septic shock, or HIV-related conditions. These findings echo prior studies that have identified metabolic dysregulation and immune dysfunction as central to the progression of acute critical illness, particularly in malnourished children^10,23,48–50^.

A key finding of this study was the identification of patient subgroups with discordant clinical and biological risk profiles. The higher mortality rate in the Biological Risk not Clinically Reflected group compared to the Clinical Risk not Biologically Reflected group highlights the limitations of current clinical and anthropometric assessments and suggests that molecular profiling could help identify high-risk patients who might otherwise be overlooked. This phenomenon has been observed in other conditions where biological signals precede obvious clinical manifestations^20,21,51^. On the other hand, the Clinical Risk not Biologically Reflected group was composed of severely undernourished children who otherwise displayed fewer signs of acute illness, suggesting an uncomplicated severe malnutrition with potential overestimation of clinical risk. Identifying these patients through clinical and biological assessments will enable improved resource allocation through earlier discharge^52,53^. The discovery that patients with high multiomic discrepancy scores had significantly worse outcomes is also noteworthy, and parallels recent critical care literature showing distinct biological subclasses of critical illness^54^.

The persistence of mortality risk signatures from admission to discharge in many patients has important implications. The similar performance of our multiomic models across hospital admission and discharge, and the high correlation between model risk scores, suggest that many patients are being discharged before achieving biological recovery. This is further supported by the strong correlation between the specific features associated with mortality. Furthermore, the presence of the same signature at discharge suggests the signature at admission is not fully driven by disease severity, highlighting the importance of addressing underlying factors in therapeutic interventions. This finding may explain the high post-discharge mortality rates observed in LMIC settings and emphasizes the clear need for more comprehensive discharge criteria and post-discharge monitoring programs^4,52,55^.

The multiomic data collected at discharge also provided evidence that treatment helped reduce the degree of biological perturbation in patients. The reductions in normalized omic distances from admission to discharge potentially indicate a partial recovery across biological systems. This is further supported by the large shift in most biological features between admission and discharge. However, some of the changes between admission and discharge were modestly associated with post-discharge mortality. Additionally, the persistent signal in the metabolome and lipidome among non-survivors points to incomplete resolution of metabolic and immune disturbances, while reduction in the metagenome omic distance could be an effect of antibiotic treatment on the microbiome. These results align with existing literature on the long-term consequences of metabolic dysfunction following critical illness, which can extend into the post-discharge period and drive mortality^10,49,56,57^.

Our analysis of the impact of anthropometric status versus age on mortality signatures provides important insights into disease biology in undernourished children. The stronger influence of nutritional status compared to age on the patients’ multiomic profiles suggests that malnutrition fundamentally alters disease response pathways in ways that immune and metabolic development with age does not. This builds on previous work showing immune dysfunction, altered metabolism, and reduced resilience to disease in malnourished children but provides a much broader view of systemic biological disruption^58–63^. Previous reports have conclusively shown that younger children, particularly infants under 12 months, are at higher risk of mortality^64,65^. Interestingly, we found that age had a relatively small impact on host perturbation within the age band recruited for this study. This finding may be explained by the narrow age enrollment boundaries and the predesigned overwhelming influence of malnutrition in this cohort, which possibly overshadowed age-related effects.

The findings from this study have important implications for clinical practice and future research. The superior performance of molecular profiling in risk stratification suggests that the development of targeted biomarker panels could improve patient triage and monitoring. The identification of distinct patient subgroups with different risk profiles could inform more personalized therapeutic approaches. In this study, we successfully distilled these biological signals to a few easily-measurable targets, paving the way to the implementation of these findings as diagnostics in low-resource. Future studies should characterize the relationship between the biological signatures and mortality (i.e., associative or causal) and interrogate the biological pathways involved at a mechanistic level. The multiomic landscape with accompanying clinical data resulting from our study also provide a rich resource, which we hope is used to evaluate future biological hypotheses. Finally, the development of practical clinical tools to define who needs specific treatments based on the distilled molecular signatures found should also be a priority.

The persistent mortality risk observed in discharged patients also emphasizes the need for improved discharge guidelines and post-discharge care strategies. Our study conclusively showed that discharged patients generally exhibit unresolved biological signals of disease that closely mirror those found at hospital admission. Recent research also supports longer-term monitoring, showing that interventions extending into the post-discharge period can significantly reduce mortality and morbidity in children recovering from severe illness^27,66^. Designing post-discharge interventions that target both nutritional deficits and unresolved biological risk will be critical to improve outcomes for discharged children.

Several limitations of this study should also be noted. First, while our study included multiple sites in different settings across six countries in Africa and South Asia, there are limitations to how generalizable these findings may be. To fully assess the implications of this study to other resource-limited settings, validation of the results presented must occur in areas with differences in types of illness, healthcare facilities and access, patient demographics, or environmental factors between sites. Simultaneously, these validation studies should explore strategies to adjust for site-related factors to improve performance across different regions. Second, while our multiomic approach was comprehensive, it needs both external validation and broadening. Given the limited sample size of the study and the limited validation performed (i.e., only proteomics), the findings presented must be validated across omics and with more samples. They may also have missed important biological signals not captured by the platforms used, such as toxins and genetic disorders.

Third, the study’s multiomic data are primarily cross-sectional, with omics collected at admission and discharge. While this enabled the comparison of biological signals at two critical time points, longitudinal omic profiling throughout hospitalization and post-discharge could provide more insights into the dynamics of disease progression and recovery. Future longitudinal studies could help identify specific time points where interventions might be most effective in reducing mortality, though we believe our data provides a refined set of parameters to look at longitudinally. Fourth, this analysis did not investigate the interplay between social factors and the biological markers of acute child illness. For maximum translational impact, it will be critical for future studies to clarify how these factors impact the progression of disease in LMICs. Finally, a major limitation of this study is the inherent barrier in therapeutic translation of its findings to under-resourced communities due to the prohibitive costs associated with multiomic profiling. Therefore, a critical avenue of future research will be on developing practical and cost-effective ways of applying the study’s findings in real-world settings.

Overall, this study provides unprecedented insights into the biological mechanisms that contribute to mortality in acutely ill children in LMICs. Our results clarify the biological basis of post-discharge mortality, underscoring the need for better discharge guidelines and post-discharge care. The identification of distinct patient subgroups and the improved understanding of the impact of malnutrition on disease progression highlight opportunities for more targeted and effective interventions. Furthermore, the strong performance of the distilled predictive models and the validation of our findings in an independent cohort demonstrate the generalizability and potential clinical utility of our work. In sum, our results will help guide the effective design of interventions aimed at reducing post-discharge mortality and improving long-term outcomes for children in resource-limited settings.

While these studies demonstrate the potential of multiomic profiling to improve health outcomes in LMICs, the integration of these data with existing clinical evaluations will be needed to develop effective and targeted therapies for vulnerable populations in low-resource settings^17^.

## Methods

### Study design

This research study complied with all relevant ethical regulations and was approved by institutional review boards of all partner sites (Oxford Tropical Research Ethics Committee, UK; the Kenya Medical Research Institute, Kenya; the University of Washington and Oregon Health and Science University, USA; Makerere University School of Biomedical Sciences Research Ethics Committee and The Uganda National Council for Science and Technology, Uganda; Aga Khan University, Pakistan; International Centre for Diarrhoeal Disease Research, (icddr,b), Bangladesh; The University of Malawi; The University of Ouagadougou and Centre Muraz, Burkina Faso; the Hospital for Sick Children, Canada; and University of Amsterdam, The Netherlands). All caregivers provided written informed consent for their child to participate in the study.

The Childhood Acute Illness and Nutrition (CHAIN) Network study recruited 3,101 children from nine sites across six countries: Bangladesh (icddr,b Dhaka Hospital and Matlab Hospital), Burkina Faso (Banfora Regional Referral Hospital), Kenya (Kilifi County Hospital, Mbagathi Sub-County Hospital, and Migori County Referral Hospital), Malawi (Queen Elizabeth Central Hospital, Blantyre), Pakistan (Civil Hospital, Karachi), and Uganda (Mulago Hospital, Kampala)^28^. Children were stratified by nutritional status using mid-upper arm circumference (MUAC) during enrollment at hospital admission, and followed up for 180 days after discharge. Geographically-matched community participants were included as a comparison group. The study was approved by the institutional review boards of all partner sites.

The study population consisted of a nested case-cohort (NCC) study (the discovery cohort) within the CHAIN cohort^11,28^. This study design, which involves randomly sampling from a larger cohort, was selected due to its ability to maximize statistical power under sample size constraints, such as the logistical and financial limitations presented by a multinational multiomic study. The discovery cohort was designed to achieve a 2:1 ratio of non-cases to cases based on analytical and simulation-based power calculations described in the published CHAIN and CHAIN NCC protocol papers^11,28^. Given the expected number of deaths in the overall cohort, it was determined that a 24% random sub-cohort plus the addition of all additional cases would yield the desired 2:1 ratio. Thus, the discovery cohort consisted of a random 24% sub-cohort of children stratified by site, which comprised 658 survivors (non-cases) and 109 deaths (cases). After the addition of all remaining deaths (241 cases) not included in the random sub-cohort, there were a total of 350 cases. Another 30 randomly selected community participants from each site (a total of 270) were also included. See the published CHAIN protocol for more details^11^.

A validation cohort of 100 children within the CHAIN study, independent of the discovery cohort, underwent proteomics profiling to assess the generalizability of the findings. Metabolomics findings were validated using admission data from the F75 case-control study^10^ among 184 children (92 inpatient deaths against 92 survivors) with complicated severe malnutrition in Kenya and Malawi.

### Biological assays

Collection and processing of all sample types were performed according to harmonized operating procedures at all study sites. Samples were collected at admission, discharge, and follow-up, and included stool, fecal swabs, whole blood, serum, plasma, and dry blood spots. Sample processing occurred under cold-chain conditions before transfer to the KEMRI Wellcome Trust Research Programme biorepository in Kilifi, Kenya. Proteomic features were generated using the SomaScan aptamer-based assay in plasma. Serum metabolomic and lipidomic features were generated using targeted and untargeted mass spectrometry techniques, respectively. Metagenomic features were generated through sequencing of DNA from stool samples. TaqMan Array Card (TAC) features were generated from nucleic acids extracted from fecal swabs. See the published CHAIN protocol for more details^11^.

### Statistical analysis and *P*-value adjustment

All analyses were performed with R (Version 4.3.1); all R packages used and their versions are available in the “Supplementary Materials”. All *p*-values were adjusted for multiple hypothesis testing using the Benjamini-Hochberg procedure^67^. Furthermore, effect sizes were also considered when interpreting the results of all hypothesis tests as an additional approach to reduce false positives.

### Model training and cross-validation

A repeated cross-validation scheme was used in all multivariate modeling to prevent overfitting and get an estimate of the performance of the model on unseen participants. In brief, participants were randomly split evenly into a training set (50%) and a test set (50%). A gradient-boosted tree (XGBoost)^29^ model was built on the training data. The model used the default parameters for classification and a maximum of 10 boosting rounds. The XGBoost model was then used to predict the outcome for the participants in the test data. This procedure was performed 25 times using different train and test sets in each iteration, and final test predictions for each participant were generated by averaging the participant’s predictions from the iterations in which the participant was present in the test set. The cross-validation framework can thus be understood as a leave-one-out cross-validation scheme with an ensemble of models trained on random samples of the training data without replacement, whose predictions are then aggregated.

The integrated multiomic model was built using a stacking architecture that generated a participant-specific weighted average of the predictions from each individual omic dataset^30^. To obtain participant-specific omic mixing weights, the iterations of the repeated cross-validation scheme in which the participant was present in the test set were extracted. Omic-specific predictions were then calculated for every other participant by averaging their predictions in only these iterations. The Wilcoxon rank-sum test was used to assess how strongly each omic separated the two classes based on the predictions from the other participants, and the -log10(Wilcoxon *p*-value) for each omic were used as the participant’s omic scores. Finally, the softmax of the participant’s omic scores was taken, with the resulting vector representing the participant-specific omic mixing weights. In particular, this stacking scheme allowed for integrated multiomic predictions to be calculated for participants with only some omics present.

Model classification performances were assessed using the area under the receiver operating characteristic curve (AUROC), the area under the precision-recall curve (AUPRC), the Wilcoxon rank-sum test, and the Lift, defined as the prevalence-adjusted AUPRC.

### Minimal model training and feature selection

The feature selection paradigm consisted of repeated subsampling of the training set to build XGBoost models in the sampled participants using the full feature set. In each iteration, the top 10 features were picked based on feature importance. After 25 iterations, the 10 multiomic features picked with the highest frequency were used together with all anthropometric features to create the minimal feature set. This minimal feature set was then used to train the minimal model on the training set and apply it to the test set. For the minimal models to classify discrepant patient subgroups, the minimal feature set consisted of the 5 multiomic features picked with the highest frequency for each of the two models, so the total number of multiomic features remained consistent. To test the minimal model on the validation set, the feature selection procedure was repeated using only features available in both the discovery and validation cohorts.

### Feature deviation scores

Feature deviation scores were defined to quantify the extent to which a specific grouping variable impacted the association of a feature with the outcome. For groupings that included both cases and controls (e.g., Anthropometric Strata and Age Group), the hedge’s *g* of the association of each feature with the outcome was calculated within the grouping of interest. For groupings that only included either cases or controls (e.g., time from hospital admission to death for cases), the hedge’s *g* of the association of each feature with the outcome was calculated using all of the participants in the other class (e.g., all controls). In both cases, the feature deviation score for a feature was defined as the standard deviation of the feature hedge’s *g* across the groupings divided by the feature hedge’s *g* across the entire dataset.

### Normalized omic distances

Normalized omic distances were defined to quantify the extent to which each participants’ feature profile was different from a normal profile, as defined using the community members. To calculate the normalized omic distance of a specific group of features, a *z*-score transformation was learned for each feature using the community members as a reference and applied to the feature values across all participants. The normalized omic distance of a specific participant, also referred to as the normalized distance from the community, was then calculated as the L2 norm of that participant’s *z*-score transformed features divided by the median L2 norm of the community members’ *z*-score transformed features.

### Contrastive analysis of the multiomic and clinical models

For the comparison between the multiomic and clinical mortality predictions, models were trained as previously described using the entire set of multiomic features or the entire set of clinical features and only participants with all omic datasets. The multiomic and clinical mortality predictions of community members were calculated to use as a reference distribution. Participants were defined as clinically or biologically sick (Clinical and Biological Scores Agree) if their predictions were above the 95th percentile of the community members’ mortality predictions, and as healthy (Clinically and Biologically Low Risk) otherwise. Participants were categorized into the discrepant groups (Biological Risk Not Clinically Reflected and Clinical Risk Not Biologically Reflected) if the distance between their multiomic and clinical predictions was above the 75th percentile.

### Multiomic discrepancy scores and contrastive interomic model analysis

Multiomic discrepancy scores were defined to quantify the extent to which each participant’s omic predictions of mortality varied from each other. To calculate the multiomic discrepancy scores, models were trained as previously described using only features from each individual omic and only participants with all omic datasets. The multiomic discrepancy score of a specific participant was then calculated as the standard deviation across all of the participant’s omic mortality predictions. For the comparison between the different omic mortality predictions, the participants with the top 10% multiomic discrepancy scores were classified as High Omic Discrepancy and the rest as Low Omic Discrepancy.

### Protein pathway analysis

Pathway enrichment was performed on the top proteomics features associated with the clinical covariates of interest. Top proteomic features were analyzed using protein set overrepresentation analysis of Gene Ontology (GO) terms^32^ performed in R with the topGO package (Version 2.42.0)^31,33^. Specifically, Fisher’s exact test was used to determine the enrichment of each GO term in the Biological Process ontology.

### Metabolite pathway analysis

Pathway analysis was performed using the Pathway Analysis module on the MetaboAnalyst 6.0 platform^34^. The background reference for the analysis consisted of the 123 detected metabolites (out of 171 metabolites measured), which could be matched to KEGG IDs using the Metabolite ID Conversion module. Most unmatched metabolites were lipids, as expected. Pathway Impact and FDR-corrected p-values were used to assess pathway enrichment.

### Lipid set enrichment analysis

Lipid set enrichment analysis was performed on the lipids measured to quantify the association of lipid characteristics, such as chain length and chain unsaturation, with the outcome of interest. Lipid features were first aggregated into four classes—phospholipids, glycerolipids, sphingolipids, and cardiolipins—and each class analyzed using lipid set enrichment analysis performed in R with the lipidr package^35,36^.

### Ethics and inclusion statement

The authors confirm that the research included local researchers throughout the research process—that is, during the design of the study, its implementation, and with respect to authorship. We also confirm that the roles and responsibilities were agreed amongst the collaborators ahead of the research and that capacity-building plans for each group of local researchers were discussed.

### Reporting summary

Further information on research design is available in the Nature Portfolio Reporting Summary linked to this article.

## Supplementary information

Supplementary Material

Reporting Summary

Transparent Peer Review File

## Source data

Source Data

## Data Availability

The data that support all the findings of this study are available within the article, the supplementary information, and the source data. Source data are provided with this paper. The data, including metadata associated with the study, are archived on the Harvard Dataverse (10.7910/DVN/X6FAGX). The data contain sensitive information about study participants and may include identifiers that could compromise confidentiality or lead to ethnic stigmatization. Access to these data requires submission of a formal request for consideration by our Data Governance Committee. Email completed data request form to the Data Governance Committee at dgc@kemri-wellcome.org. The requester provides investigators with details, variables requested, intended use of the dataset, potential risks of the study, including risks to confidentiality of individuals or communities, potential benefits of the study, including to participant communities, scientific capacity building or health policy and planned outputs (if analysis on the dataset will result in publication or reports or presentations). The requester also needs to formally agree to the conditions and limitations for data sharing to avoid misuse of shared data. Processing of data requests takes between 4 weeks to 6 weeks from submission. The raw metagenomic sequencing data of the CHAIN NCC study have been deposited at the European Nucleotide Archive (ENA) under accession number ERP187936. Source data are provided with this paper.

## Code availability

The analysis code that support the findings of this study are archived and publicly available on the Harvard Dataverse (10.7910/DVN/X6FAGX) and on Dryad (10.5061/dryad.p2ngf1w1z).
