# Supplementary material for: Multiomics characterization of acute child illness and mortality in Africa and South Asia
Source: Nat Commun. 2026 Apr 13;17:5171. doi: 10.1038/s41467-026-69754-w (PMC13249890; doi:10.1038/s41467-026-69754-w)
Supplement: Supplementary file 1 — Supplementary Material [file 41467_2026_69754_MOESM1_ESM.pdf]

Supplementary Figures

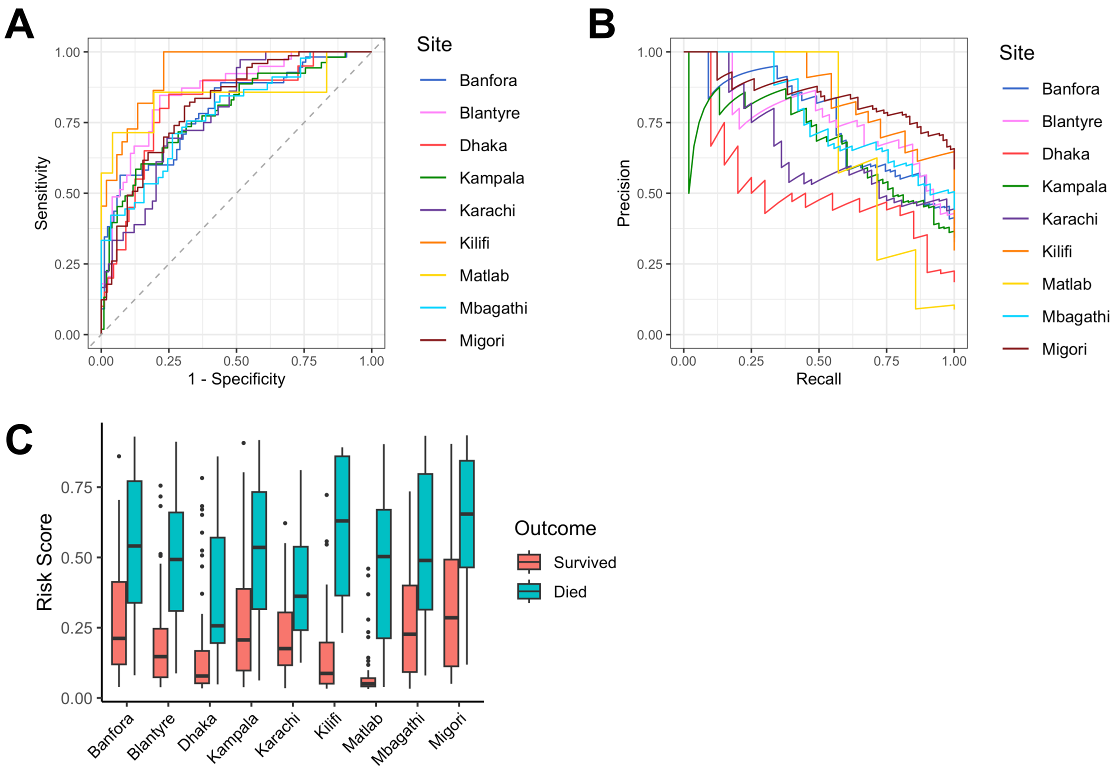

Figure S1. Performance of the multiomic model for the prediction of mortality during hospitalization and post-discharge. A cross-validated XGBoost model for the prediction of mortality during hospitalization or in the post-discharge period was trained on the integrated multiomic data of the discovery cohort. (**A**) ROC curves for the multiomic model stratified by study site. (**B**) Precision-recall curves for the multiomic model stratified by study site. (**C**) Distribution of the mortality risk scores predicted by the multiomic model stratified by patient outcome and study site. Box plots indicate median (middle line); 25th and 75th percentiles (box limits); 1.5*interquartile range (error bars); and outliers (single points).

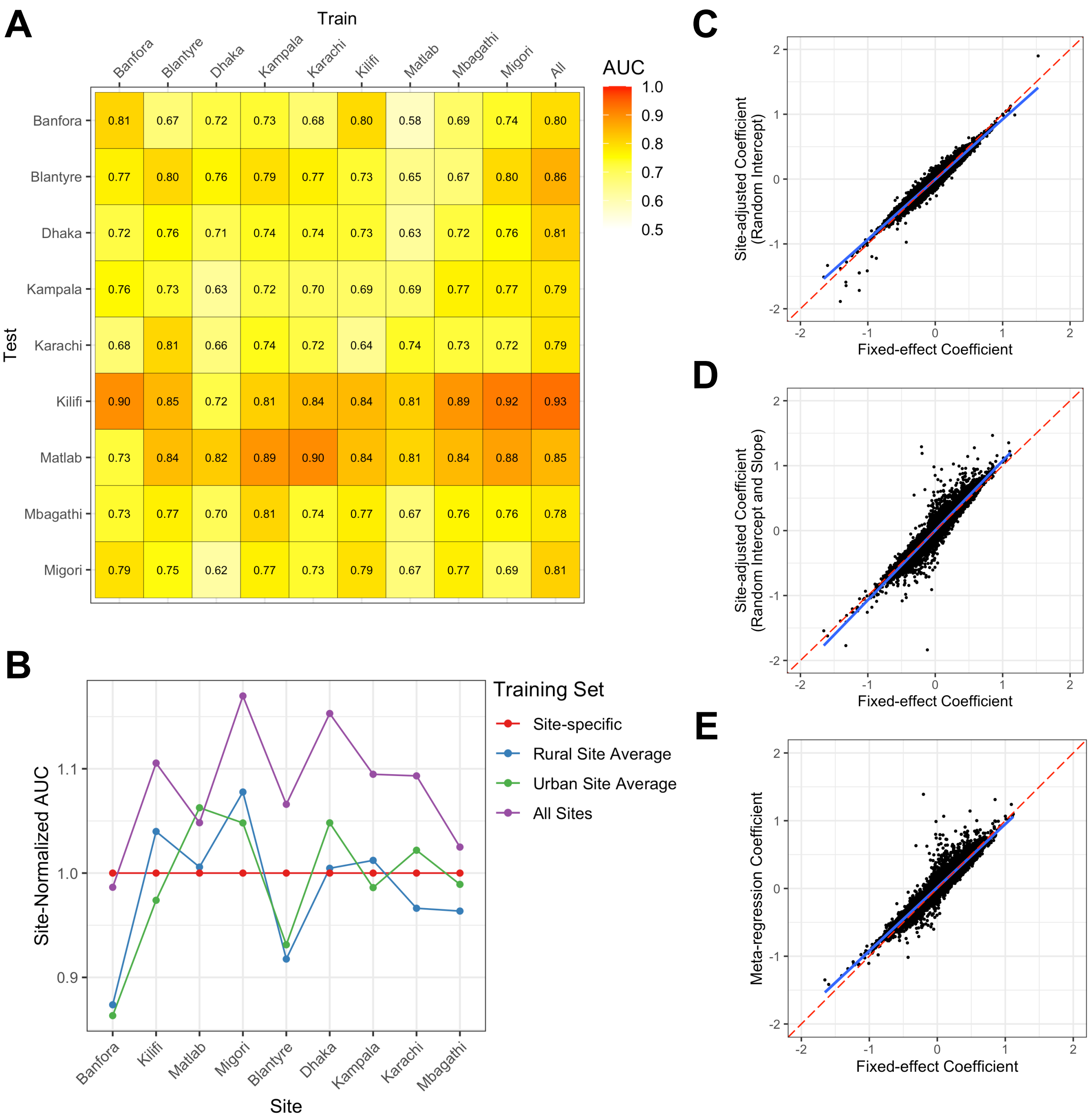

Figure S2. Generalizability of the multiomic signatures of mortality across sites. (**A**-**B**) Site-specific cross-validated XGBoost models were built for the prediction of mortality during hospitalization or in the post-discharge period. (**A**) Classification performance of each site-specific model assessed across every other site using the AUROC. (**B**) Site-normalized AUROCs for site-specific models applied across every other site and aggregated by urban or rural status. (**C**-**E**) Each multiomic feature's association with mortality at hospital admission was quantified with site-unadjusted and site-adjusted logistic regressions. Non-adjusted fixed-effect coefficient estimates are shown compared to site-adjusted coefficient estimates generated with random intercepts (**C**), random intercepts and random slopes (**D**), or a meta-regression framework (**E**). The blue lines and shadows represent the regression lines and the 95% Confidence Intervals, respectively.

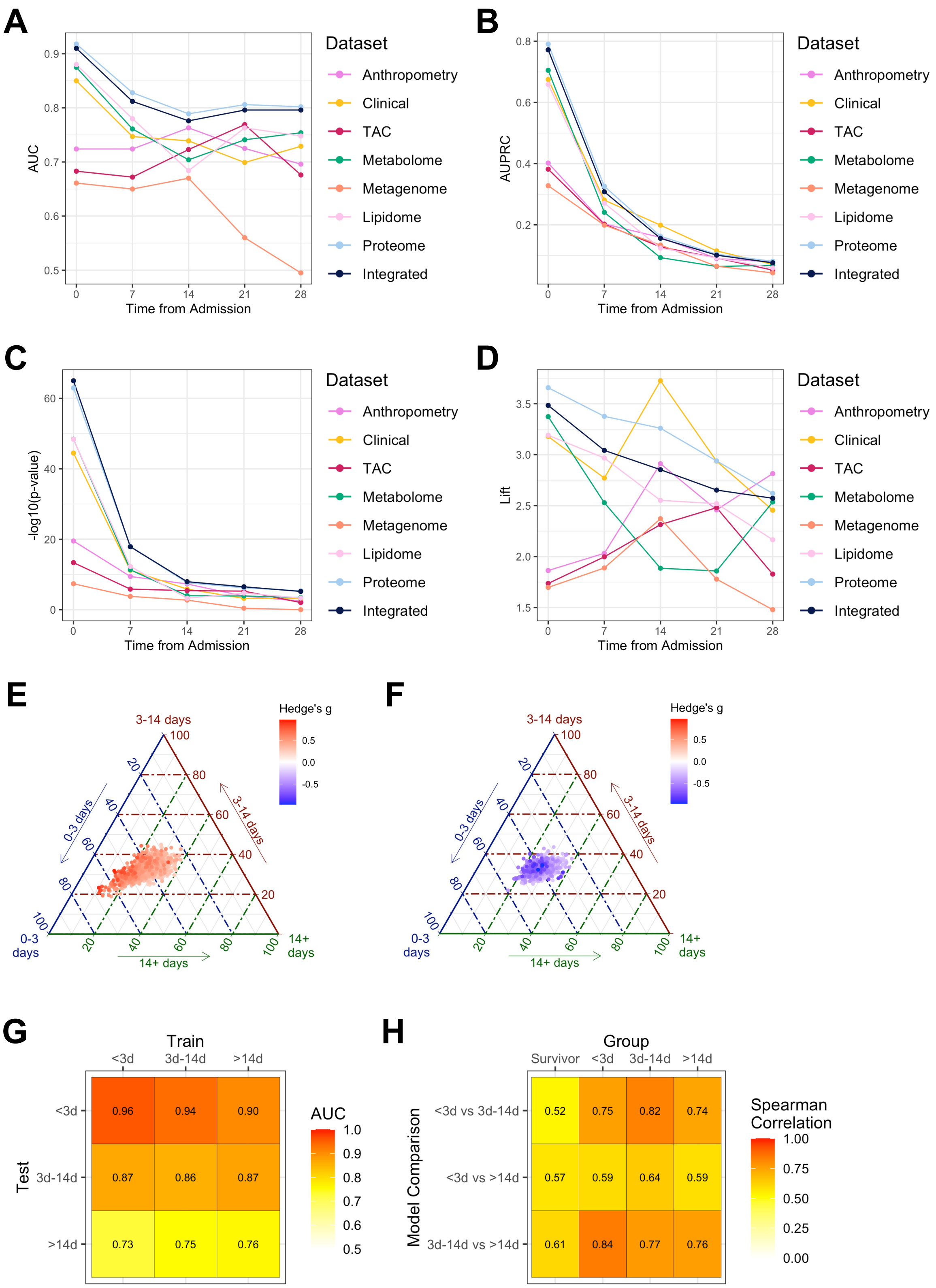

A
B
C
D
E
F
G
H
Dataset
Anthropometry
Clinical
TAC
Metabolome
Metagenome
Lipidome
Proteome
Integrated
AUC
AUPRC
-log10(p-value)
Lift
Time from Admission
Hedge's g
3-14 days
0-3 days
14+ days
Train
Test
AUC
Group
Model Comparison
Spearman Correlation
Survivor
<3d
3d-14d
>14d
<3d vs 3d-14d
<3d vs >14d
3d-14d vs >14d
0.96
0.94
0.90
0.87
0.86
0.87
0.73
0.75
0.76
0.52
0.75
0.82
0.74
0.57
0.59
0.64
0.59
0.61
0.84
0.77
0.76

Figure S3. Temporal analysis of the multiomic signature of mortality during hospitalization and post-discharge. (**A-D**) Cross-validated XGBoost models were built using each dataset separately for the prediction of mortality during hospitalization or in the post-discharge period and their performances assessed using only the cases that occurred in 14-day intervals. AUROCs (**A**), AUPRCs (**B**), uncorrected Wilcoxon rank sum test p-values (**C**), and lifts (prevalence-adjusted AUPRCs, **D**) of the models built on each dataset evaluated on 14-day intervals. (**E-F**) The hedge's g for each feature's association with mortality or survival at hospital admission was calculated using only cases that died in the specified timeframe. Ternary plots depicting each timeframe's relative hedge's g for each feature significantly associated with mortality (**E**) or survival (**F**) with at least a small effect size (|hedge's g| > 0.2. (**G-H**) Cross-validated XGBoost models for the prediction of mortality during hospitalization or in the post-discharge period were built using only the cases that occurred in the specified timeframes. Models were then tested with the cases from the other timeframes (**G**) and model predictions between models compared (**H**).

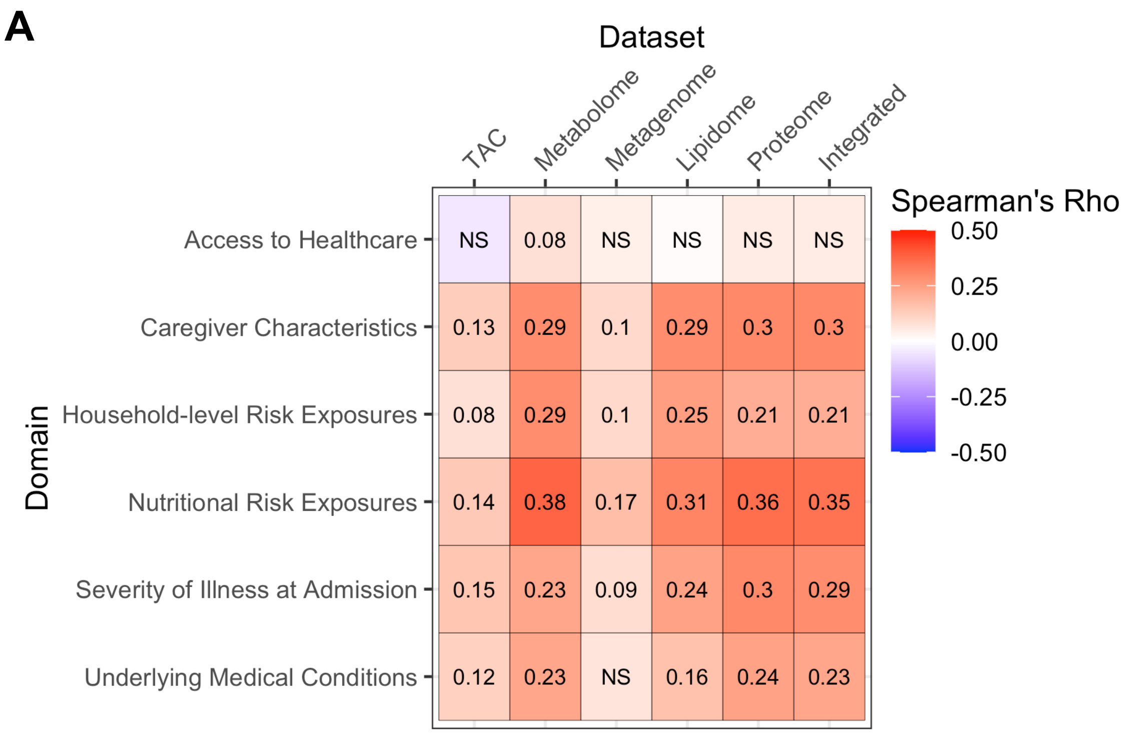

Figure S4. Association of the multiomic signatures of mortality and immediate and underlying domains of exposure. Cross-validated XGBoost models were built using each dataset separately for the prediction of mortality during hospitalization or in the post-discharge period. Immediate and underlying exposure variables influencing mortality risk were grouped into domains to generate latent factors capturing overall exposure effects. (**A**) Heatmap showing the Pearson correlation of the different omic signatures of mortality with different domains of exposure. NS = Not Significant.

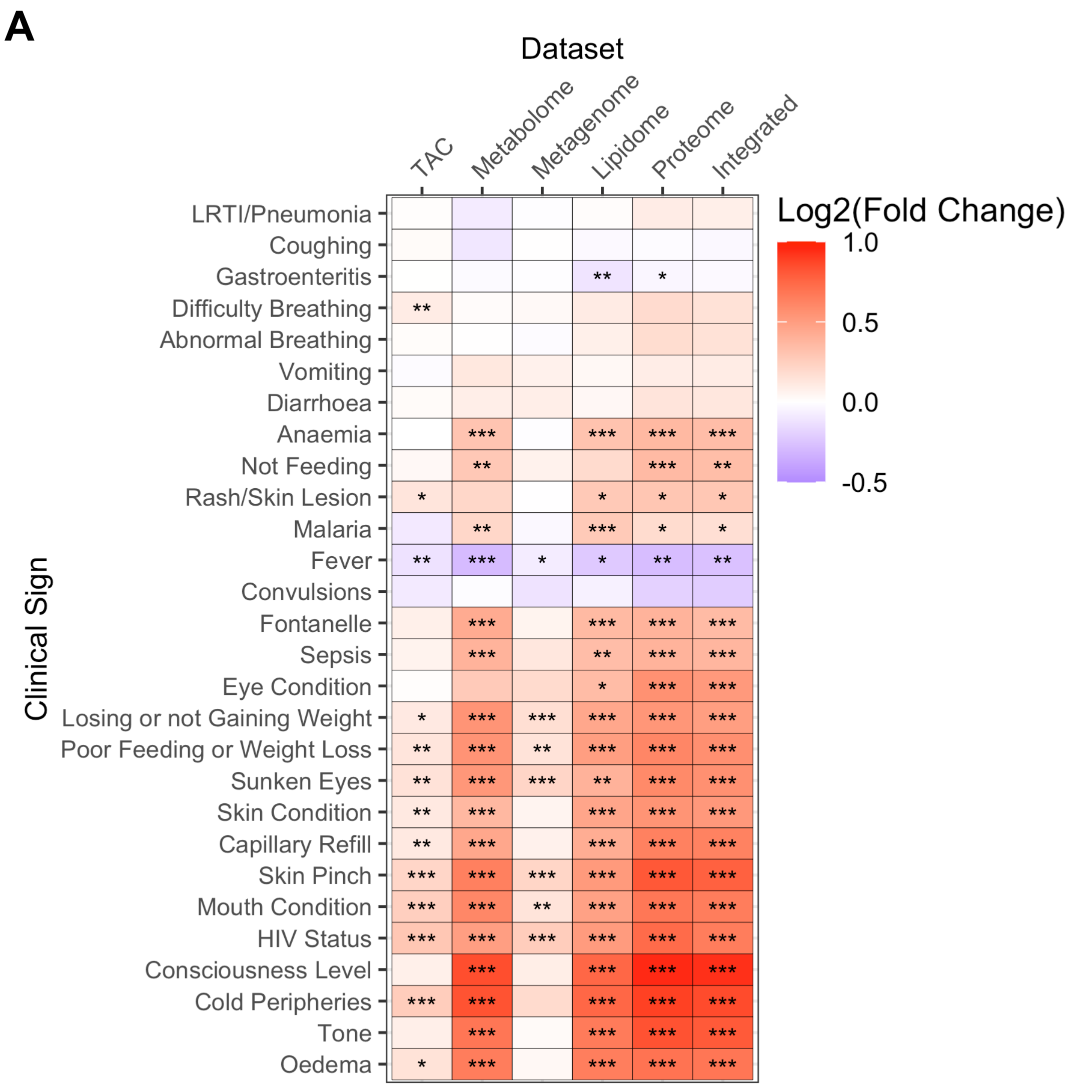

Figure S5. Association of the multiomic signatures of mortality and clinical signs of disease at admission. Cross-validated XGBoost models were built using each dataset separately for the prediction of mortality during hospitalization or in the post-discharge period. (**A**) Heatmap showing the association of the different omic signatures of mortality with different clinical signs of disease. Asterisks indicate statistical significance assessed with a two-tailed Wilcoxon rank sum test and corrected for multiple hypothesis testing: *$p < 0.05$, **$p < 0.01$, ***$p < 0.001$.

A

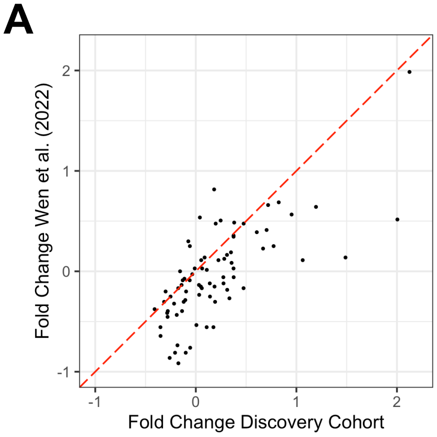

Figure S6. Validation of the metabolomic signature of mortality with the F75 study. Metabolites between studies were matched using Human Metabolome Database IDs. The association with mortality of each matched metabolite was assessed in the discovery cohort using log fold change. (**A**) Comparison of the association of each metabolite with mortality in the discovery cohort and the previously-published metabolomic results from the F75 study.

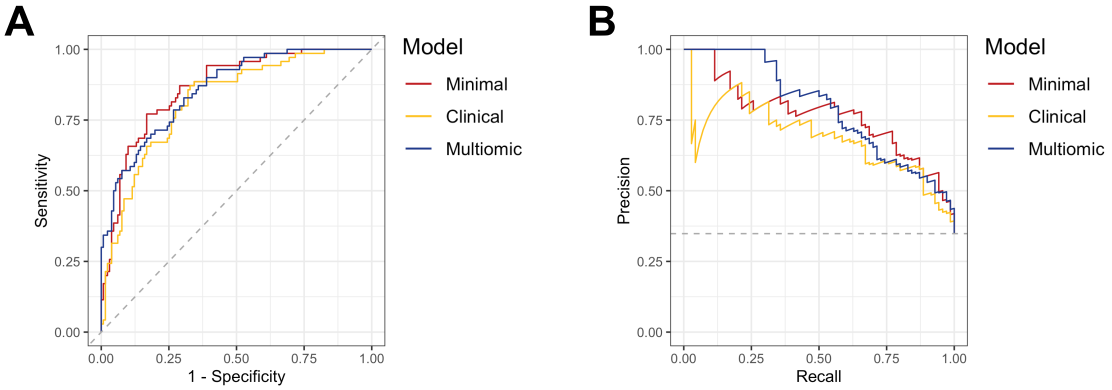

Figure S7. Performance of the minimal model for the prediction of mortality during hospitalization and post-discharge. Participants were randomly split into a training set (N = 807, 80%) and a test set (N = 201, 20%) to build a minimal XGBoost model for the prediction of mortality. ROC (**A**) and precision-recall (**B**) curves for the minimal, multiomic, and clinical models.

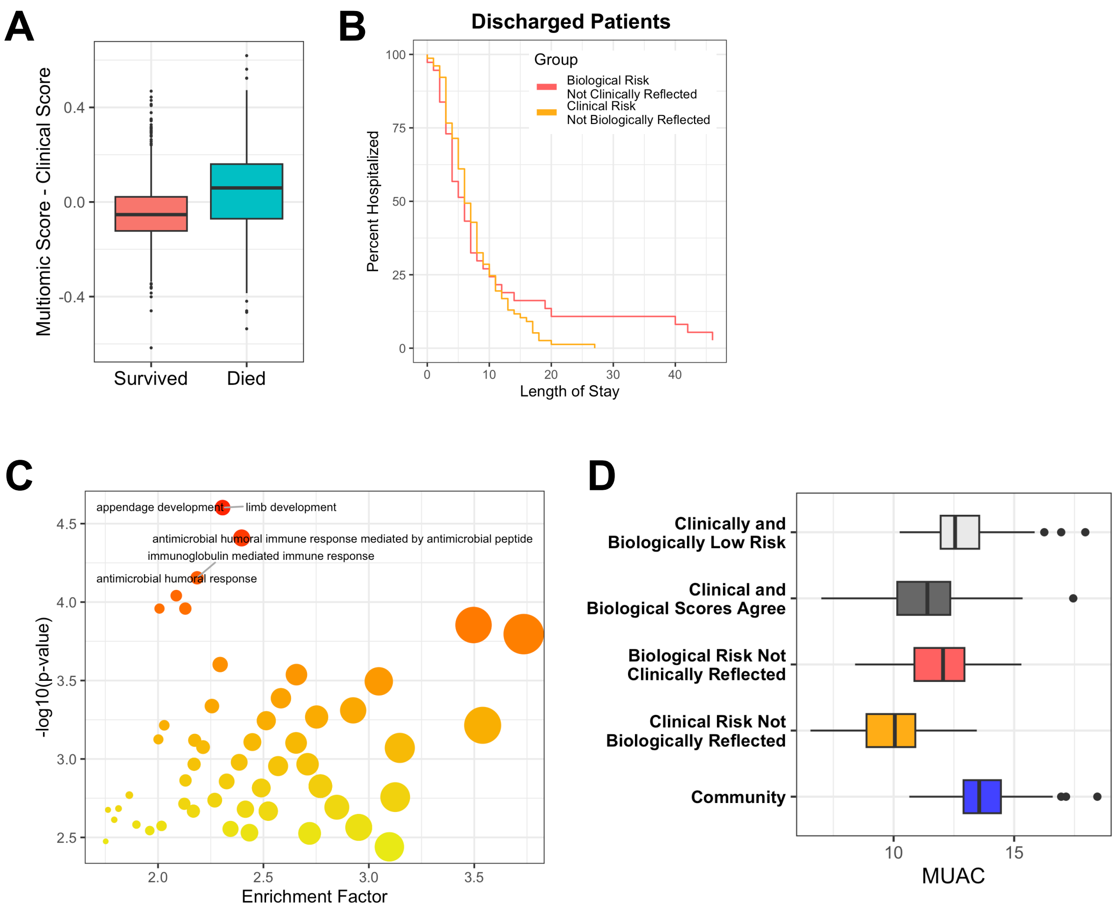

Figure S8. Characterization of the patient subgroups with discrepant clinical and multiomic mortality risk scores at hospital admission. (**A**) The differences between the mortality risk scores predicted by the multiomic model and the clinical model described in **Figure 2B** stratified by patient outcome ($N = 965$). (**B-D**) Cross-validated XGBoost models for the prediction of mortality during hospitalization or in the post-discharge period were trained on the clinical data and the integrated multiomic data for the subset of discovery cohort members with all omics datasets available ($N = 629$). (**B**) Kaplan-Meier curve for the discrepant subgroups defined in **Figure 3A** showing the length of hospital stay for all discharged patients. (**C**) GO overrepresentation analysis performed on the plasma proteome between the discrepant subgroups defined in **Figure 3A**. Plot shows uncorrected two-sided p-values obtained using Fisher's exact test. (**D**) Distribution of patient MUACs stratified by the patient subgroups defined in **Figure 3A**. Box plots indicate median (middle line); 25th and 75th percentiles (box limits); 1.5*interquartile range (error bars); and outliers (single points).

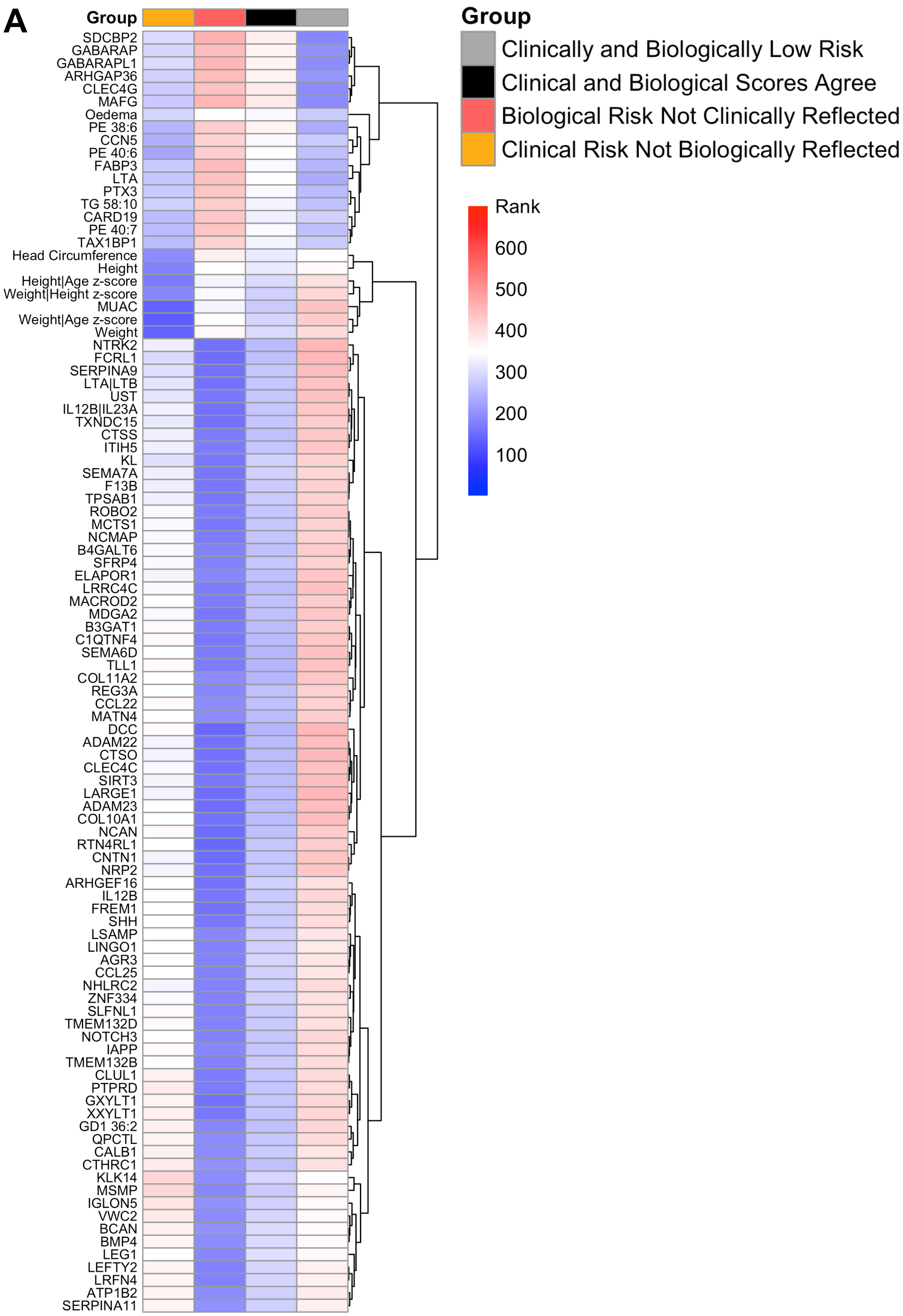
A
Group
SDCBP2
GABARAP
GABARAPL1
ARHGAP36
CLEC4G
MAFG
Oedema
PE 38:6
CCN5
PE 40:6
FABP3
LTA
PTX3
TG 58:10
CARD19
PE 40:7
TAX1BP1
Head Circumference
Height
Height|Age z-score
Weight|Height z-score
MUAC
Weight|Age z-score
Weight
NTRK2
FCRL1
SERPINA9
LTA|LTB
UST
IL12B|IL23A
TXNDC15
CTSS
ITIH5
KL
SEMA7A
F13B
TPSAB1
ROBO2
MCTS1
NCMAP
B4GALT6
SFRP4
ELAPOR1
LRRC4C
MACROD2
MDGA2
B3GAT1
C1QTNF4
SEMA6D
TLL1
COL11A2
REG3A
CCL22
MATN4
DCC
ADAM22
CTSO
CLEC4C
SIRT3
LARGE1
ADAM23
COL10A1
NCAN
RTN4RL1
CNTN1
NRP2
ARHGEF16
IL12B
FREM1
SHH
LSAMP
LINGO1
AGR3
CCL25
NHLRC2
ZNF334
SLFNL1
TMEM132D
NOTCH3
IAPP
TMEM132B
CLUL1
PTPRD
GXYLT1
XXYLT1
GD1 36:2
QPCTL
CALB1
CTHRC1
KLK14
MSMP
IGLON5
VWC2
BCAN
BMP4
LEG1
LEFTY2
LRFN4
ATP1B2
SERPINA11
Group
Clinically and Biologically Low Risk
Clinical and Biological Scores Agree
Biological Risk Not Clinically Reflected
Clinical Risk Not Biologically Reflected
Rank
600
500
400
300
200
100

Figure S9. Contrasting features of the patient subgroups with discrepant clinical and multiomic mortality risk scores at hospital admission. Cross-validated XGBoost models for the prediction of mortality during hospitalization or in the post-discharge period were trained on the clinical data and the integrated multiomic data for the subset of discovery cohort members with all omics datasets available (*N* = 629). (**A**) Heatmap of the clinical and multiomic features most different between the discrepant subgroups defined in **Figure 3A**.

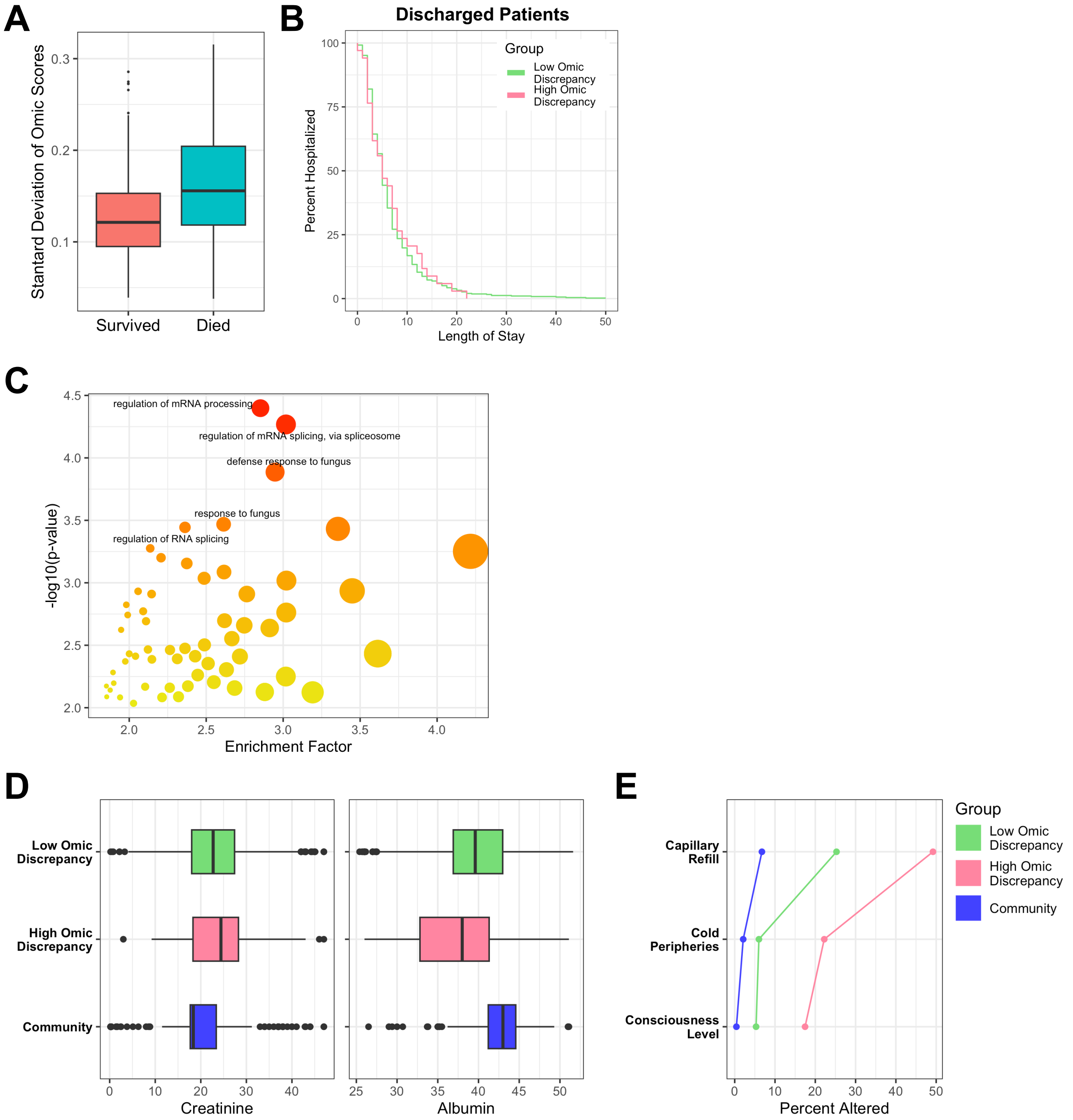

Figure S10. Characterization of the patient subgroups with discrepant interomic mortality risk scores at hospital admission. (**A**) Multiomic discrepancy scores of the models in **Figure 2B** stratified by patient outcome ($N = 629$). (**B-E**) Cross-validated XGBoost models for the prediction of mortality during hospitalization or in the post-discharge period were trained on each omic dataset for the subset of discovery cohort members with all omics datasets available ($N = 629$). (**B**) Kaplan-Meier curve for the discrepant subgroups defined in **Figure 3E** showing the length of hospital stay for all discharged patients. (**C**) GO overrepresentation analysis performed on the plasma proteome between the discrepant subgroups defined in **Figure 3E**. Plot shows uncorrected two-sided p-values obtained using Fisher's exact test. (**D**) Distribution of

patient creatinine and albumin levels stratified by the patient subgroups defined in **Figure 3E**. (**E**) Percentage of patients with altered clinical assessments stratified by the patient subgroups defined in **Figure 3E**. Box plots indicate median (middle line); 25th and 75th percentiles (box limits); 1.5*interquartile range (error bars); and outliers (single points).

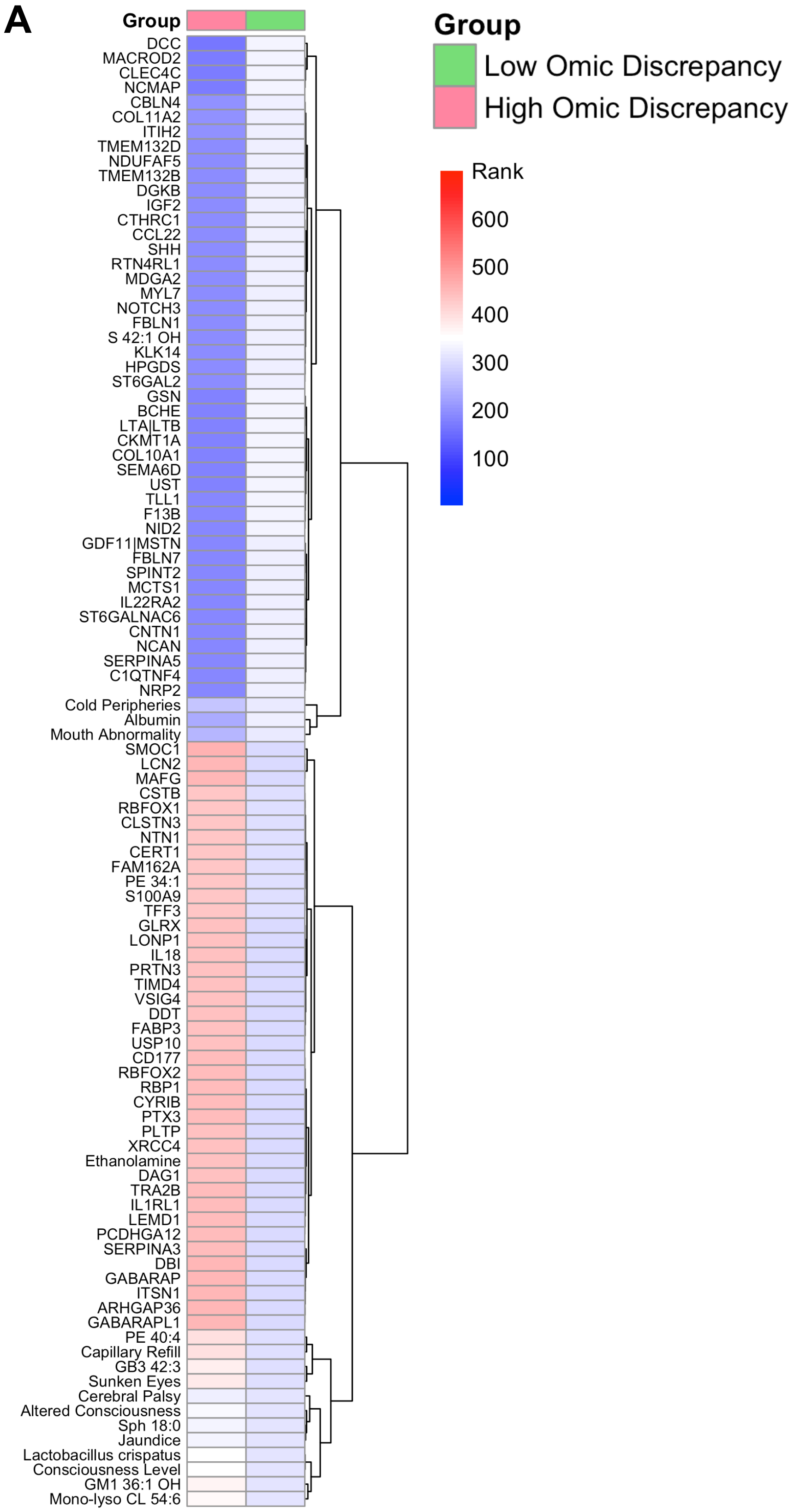
A
Group
Low Omic Discrepancy
High Omic Discrepancy
Rank
600
500
400
300
200
100
DCC
MACROD2
CLEC4C
NCMAP
CBLN4
COL11A2
ITIH2
TMEM132D
NDUFAF5
TMEM132B
DGKB
IGF2
CTHRC1
CCL22
SHH
RTN4RL1
MDGA2
MYL7
NOTCH3
FBLN1
S 42:1 OH
KLK14
HPGDS
ST6GAL2
GSN
BCHE
LTA|LTB
CKMT1A
COL10A1
SEMA6D
UST
TLL1
F13B
NID2
GDF11|MSTN
FBLN7
SPINT2
MCTS1
IL22RA2
ST6GALNAC6
CNTN1
NCAN
SERPINA5
C1QTNF4
NRP2
Cold Peripheries
Albumin
Mouth Abnormality
SMOC1
LCN2
MAFG
CSTB
RBFOX1
CLSTN3
NTN1
CERT1
FAM162A
PE 34:1
S100A9
TFF3
GLRX
LONP1
IL18
PRTN3
TIMD4
VSIG4
DDT
FABP3
USP10
CD177
RBFOX2
RBP1
CYRIB
PTX3
PLTP
XRCC4
Ethanolamine
DAG1
TRA2B
IL1RL1
LEMD1
PCDHGA12
SERPINA3
DBI
GABARAP
ITSN1
ARHGAP36
GABARAPL1
PE 40:4
Capillary Refill
GB3 42:3
Sunken Eyes
Cerebral Palsy
Altered Consciousness
Sph 18:0
Jaundice
Lactobacillus crispatus
Consciousness Level
GM1 36:1 OH
Mono-lyso CL 54:6

Figure S11. Contrasting features of the patient subgroups with discrepant interomic mortality risk scores at hospital admission. Cross-validated XGBoost models for the prediction of mortality during hospitalization or in the post-discharge period were trained on each omic dataset for the subset of discovery cohort members with all omics datasets available ($N = 629$). (**A**) Heatmap of the clinical and multiomic features most different between the discrepant subgroups defined in **Figure 3E**.

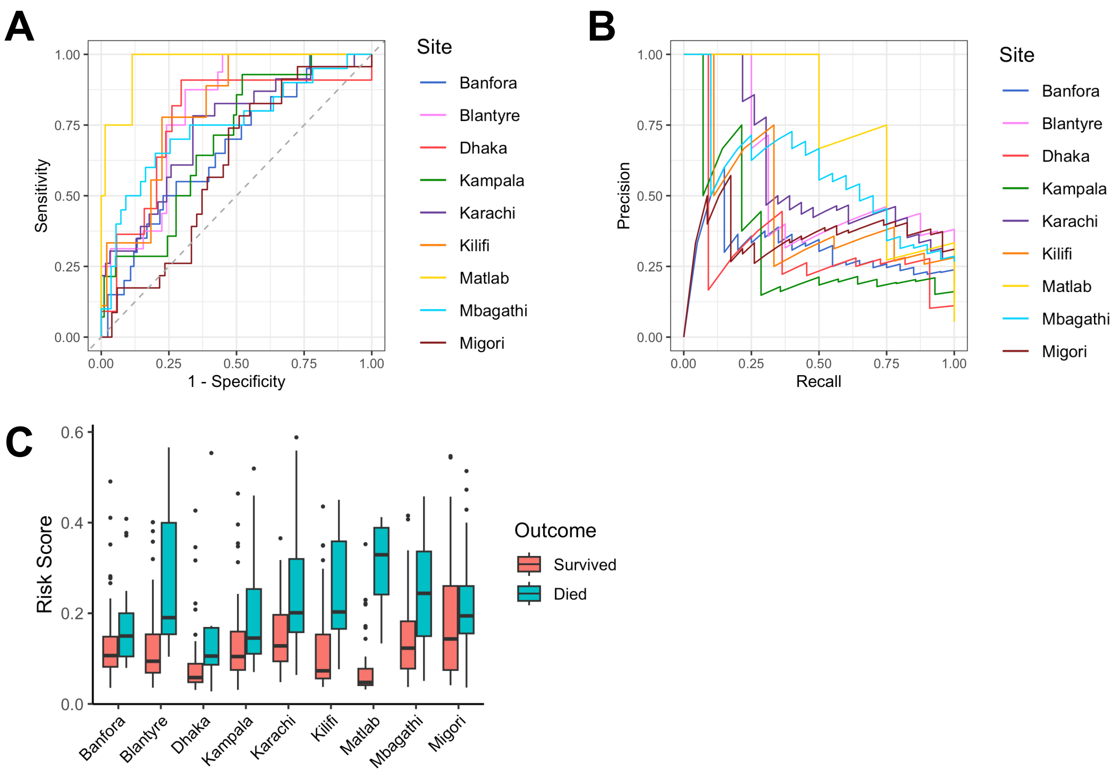

Figure S12. Site-specific analysis of the multiomic model for the prediction of mortality in the post-discharge period. A cross-validated XGBoost model for the prediction of mortality in the post-discharge period was trained on the integrated multiomic data of the discovery cohort. (**A**) ROC curves for the multiomic model stratified by site of origin. (**B**) Precision-recall curves for the multiomic model stratified by site of origin. (**C**) Distribution of the mortality risk scores predicted by the multiomic model stratified by patient outcome and site of origin. Box plots indicate median (middle line); 25th and 75th percentiles (box limits); 1.5*interquartile range (error bars); and outliers (single points).

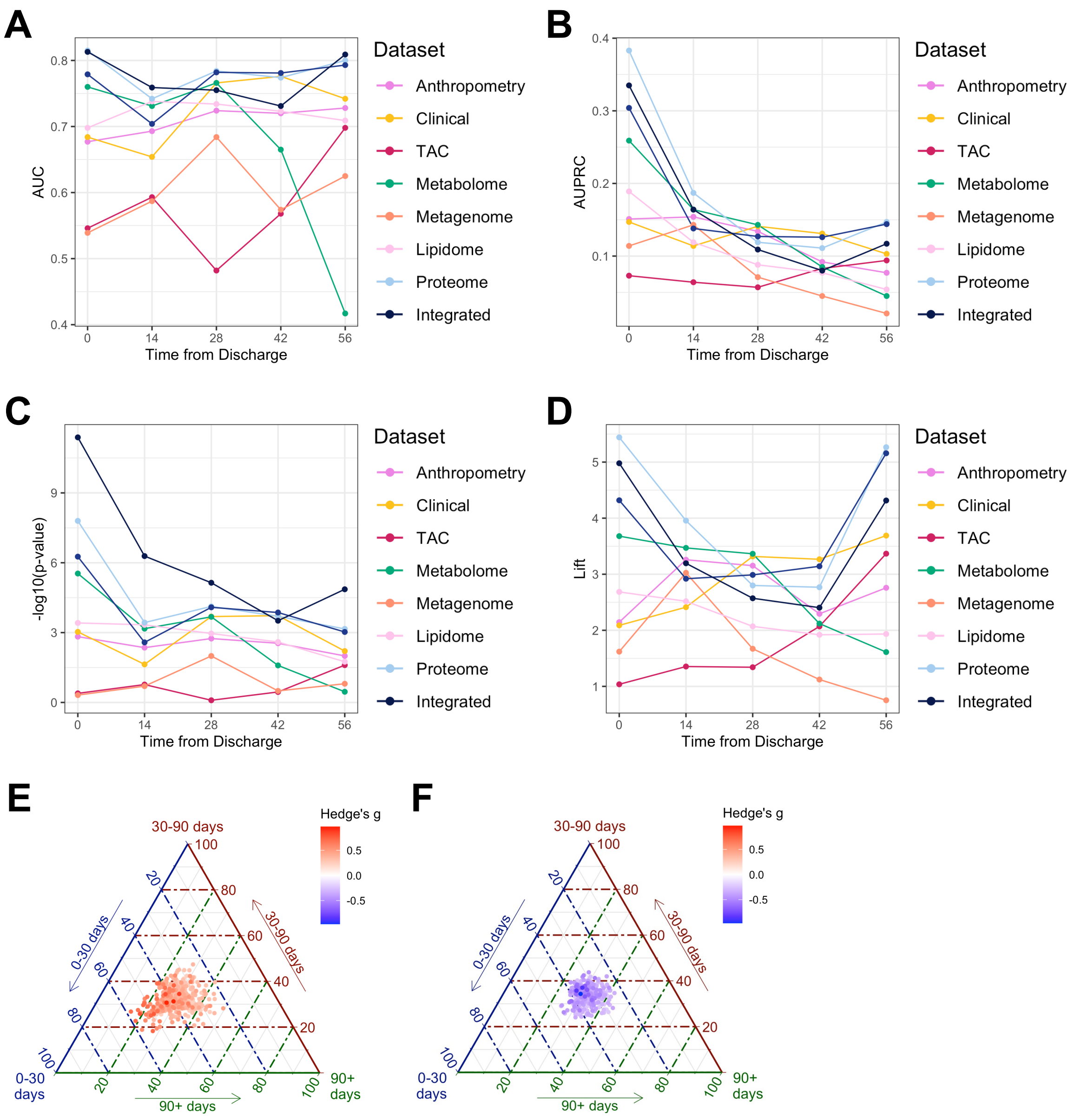

Figure S13. Temporal analysis of the multiomic signature of mortality in the post-discharge period. (**A-D**) Cross-validated XGBoost models were built using each dataset separately for the prediction of mortality in the post-discharge period and their performances assessed using only the cases that occurred in 14-day intervals. AUROCs (**A**), AUPRCs (**B**), unadjusted two-tailed Wilcoxon p-values (**C**), and lifts (prevalence-adjusted AUPRCs, **D**) of the models built on each dataset evaluated on 14-day intervals. (**E-F**) The hedge's g for each feature's association with mortality or survival at discharge was calculated using only cases that died in the specified timeframe. Ternary plots depicting each timeframe's relative hedge's g for each feature significantly associated with mortality (**E**) or survival (**F**) with at least a small effect size (|hedge's g| > 0.2.

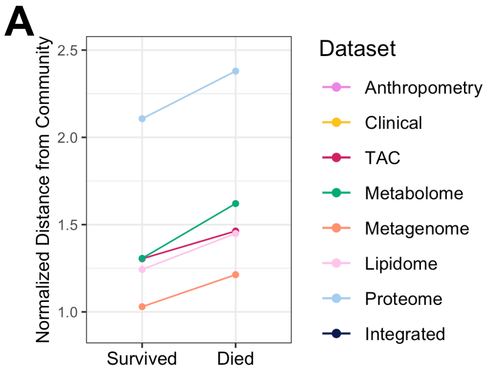

Figure S14. Multiomic diversity at discharge. (**A**) Median normalized distance from the community at discharge stratified by outcome for each omic dataset analyzed**.**

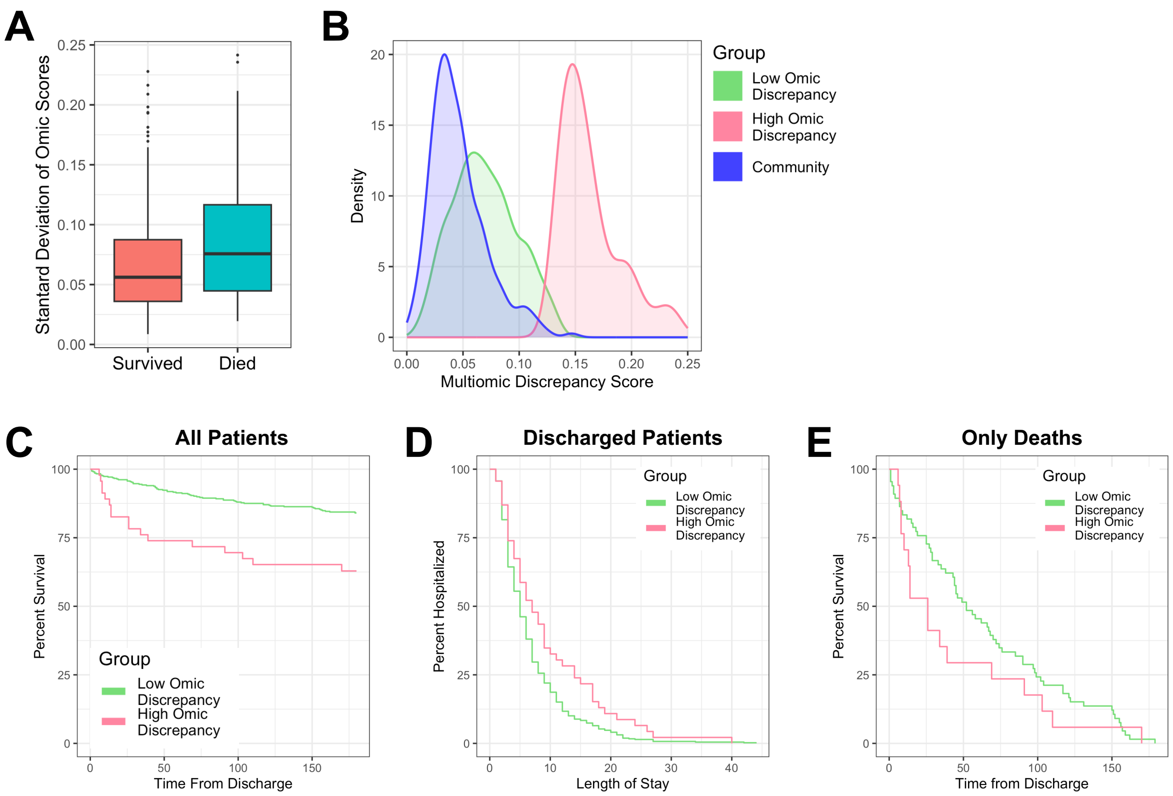

Figure S15. Characterization of the patient subgroups with discrepant interomic mortality risk scores at discharge. (**A**) Multiomic discrepancy scores of the models in **Figure 4B** stratified by patient outcome (*N* = 466). (**B-E**) Cross-validated XGBoost models for the prediction of mortality in the post-discharge period were trained on each omic dataset for the subset of discovery cohort members with all omics datasets available at discharge (*N* = 466). The risk scores of community members based on these models were then calculated. (**B**) Distribution of multiomic discrepancy scores, with colors representing patient subgroups. (**C**) Kaplan-Meier survival curves for the discrepant subgroups defined in **B** showing the survival curves based on all patients. (**D**) Kaplan-Meier curve for the discrepant subgroups defined in **B** showing the length of hospital stay for all discharged patients. (**E**) Kaplan-Meier survival curves for the discrepant subgroups defined in **B** showing the survival curve based on only patients that died. Box plots indicate median (middle line); 25th and 75th percentiles (box limits); 1.5*interquartile range (error bars); and outliers (single points).

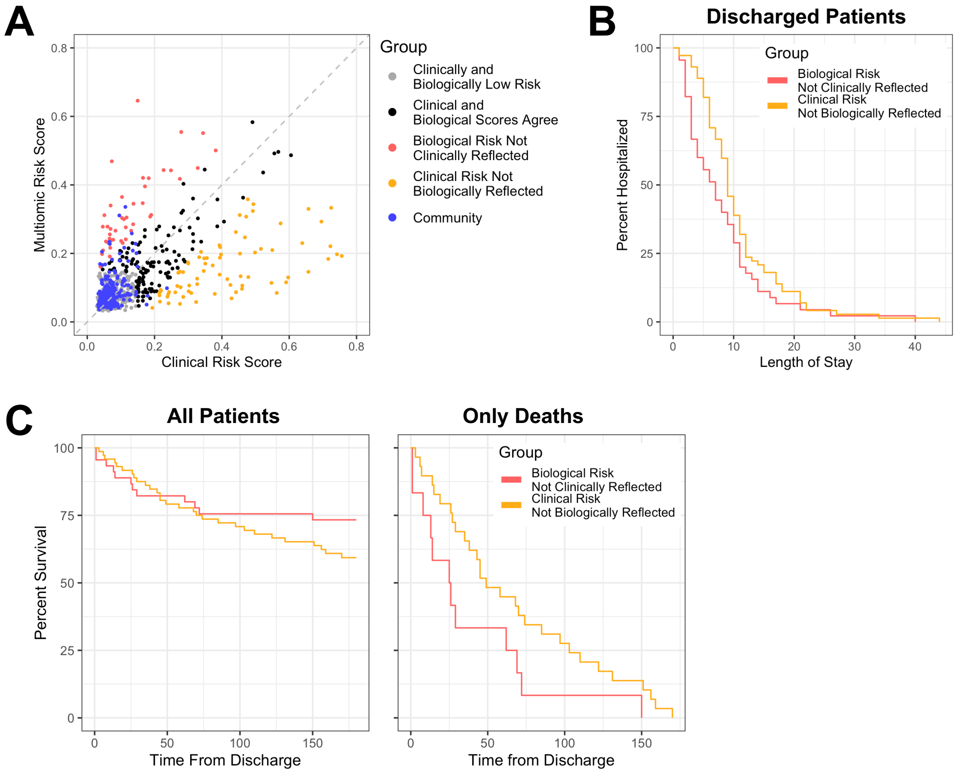

Figure S16. Contrastive analysis of the multiomic and clinical models of mortality at discharge. Cross-validated XGBoost models for the prediction of mortality in the post-discharge period were trained on the clinical data and the integrated multiomic data for the subset of discovery cohort members with all omics datasets available at discharge ($N = 466$). The risk scores of community members based on these models were then calculated. (**A**) Comparison between the mortality risk scores predicted by the multiomic and the clinical models, with colors representing patient subgroups. (**B**) Kaplan-Meier curve for the discrepant subgroups defined in **A** showing the length of hospital stay for all discharged patients. (**C**) Kaplan-Meier survival curves for the discrepant subgroups defined in **A** showing the survival curves based on all patients (left) or only on patients that died (right).

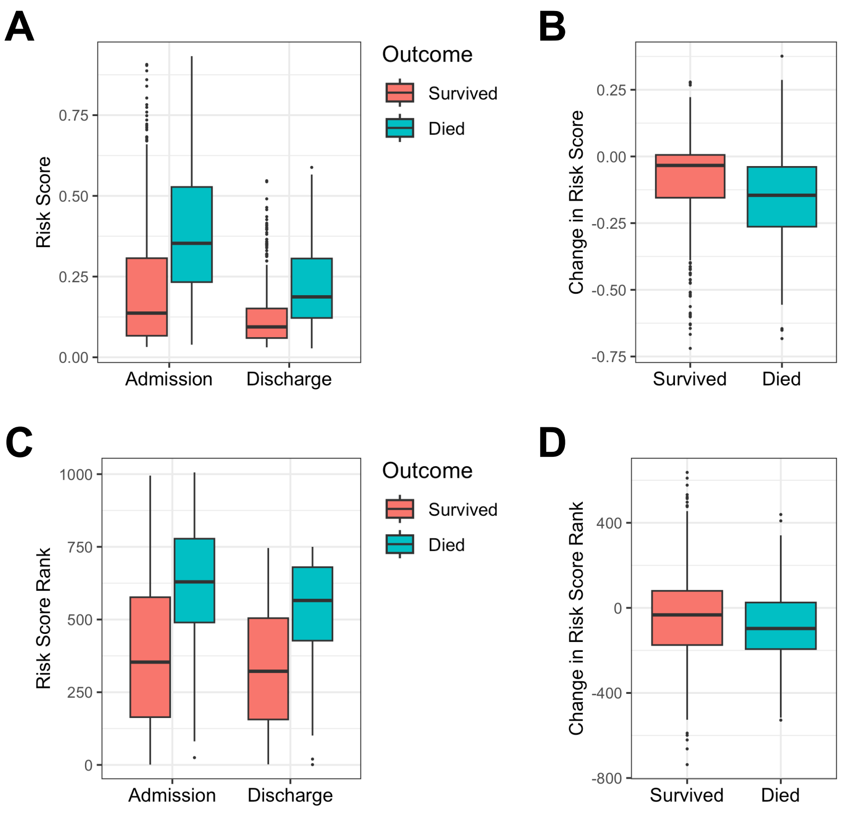

Figure S17. Comparison of the multiomic mortality risk scores between hospital admission and discharge. Cross-validated XGBoost models were built on the integrated multiomic data for the prediction of mortality during hospitalization or in the post-discharge period using data collected at hospital admission or for the prediction of mortality in the post-discharge period using data collected at discharge. (**A**) Multiomic mortality risk scores stratified by outcome and timepoint. (**B**) Change in multiomic mortality risk scores stratified by outcome. (**C**) Multiomic mortality risk score rank stratified by outcome and timepoint. (**D**) Change in multiomic mortality risk score rank stratified by outcome. Box plots indicate median (middle line); 25th and 75th percentiles (box limits); 1.5*interquartile range (error bars); and outliers (single points).

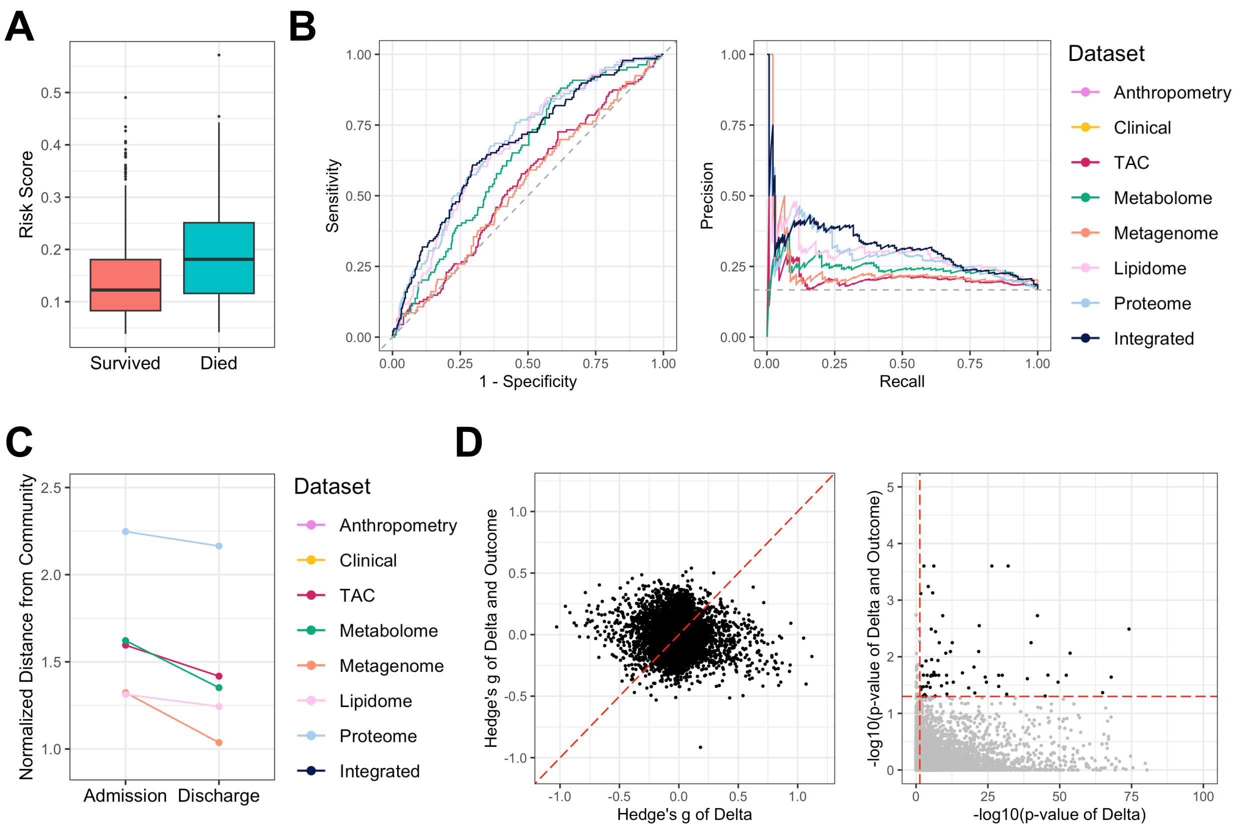

Figure S18. Impact of hospitalization on the multiomic signatures of mortality at discharge. The change in each multiomic feature was calculated for all patients with data at both admission and discharge. An XGBoost model for the prediction of mortality in the post-discharge period was trained on the integrated multiomic change data. (**A**) Distribution of the mortality risk scores predicted by the multiomic change model stratified by patient outcome. (**B**) Cross-validated XGBoost models were built using each change dataset separately for the prediction of mortality in the post-discharge period. Left: Receiver-operating characteristic curves for the multiomic change model and the models built on each change dataset. Right: Precision-recall curve for the multiomic change model and the models built on each change dataset. (**C**) Median normalized distance from the community at hospital admission and discharge for each dataset analyzed. (**D**) Comparison of the change in multiomic features between admission and discharge and the association of this change to post-discharge mortality, assessed using effect size (Left) and FDR-adjusted statistical significance assessed using two-tailed Wilcoxon signed-rank test (Right). Box plots indicate median (middle line); 25th and 75th percentiles (box limits); 1.5*interquartile range (error bars); and outliers (single points).

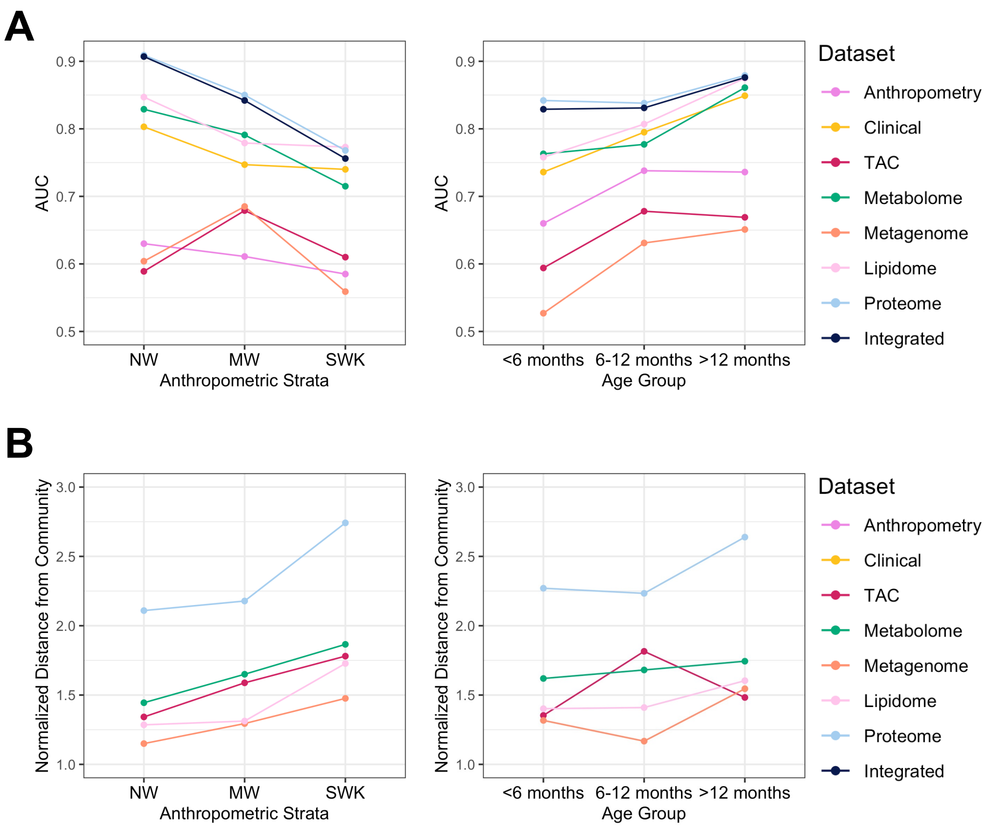

Figure S19. Characterizing the impact of anthropometric strata and age on the multiomic signature of mortality at hospital admission. Cross-validated XGBoost models for the prediction of mortality during hospitalization or in the post-discharge period of the discovery cohort were trained on the data collected at hospital admission. (**A**) Model performances stratified by anthropometric strata (left) and age group (right). (**B**) Median normalized distance from the community stratified by anthropometric strata and age group for each omic dataset analyzed and the integrated multiomic data. NW = Not Wasted, MW = Moderately Wasted, and SWK = Severely Wasted or Kwashiorkor.

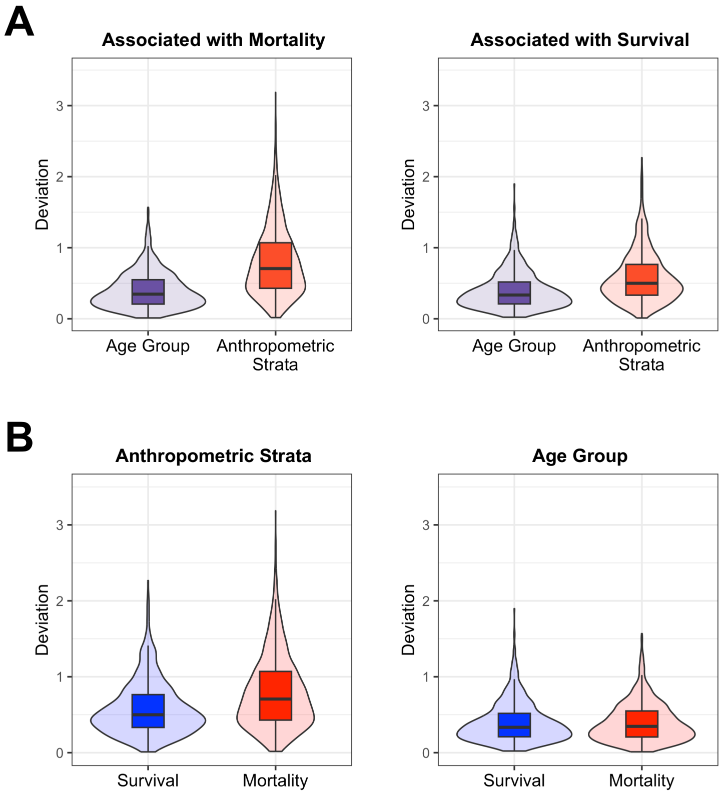

Figure S20. Feature associations with mortality and survival within anthropometric strata and age groups at hospital admission. Feature deviation scores within anthropometric strata and age groups were calculated at hospital admission for each feature significantly associated with mortality or survival with at least a small effect size (|hedge's g| > 0.2 (**A**) The distribution of feature deviation scores at hospital admission by association with mortality (left) or survival (right). (**B**) The distribution of feature deviation scores at hospital admission by anthropometric strata (left) and age group (right). Box plots indicate median (middle line); 25th and 75th percentiles (box limits); 1.5*interquartile range (error bars); and outliers (single points).

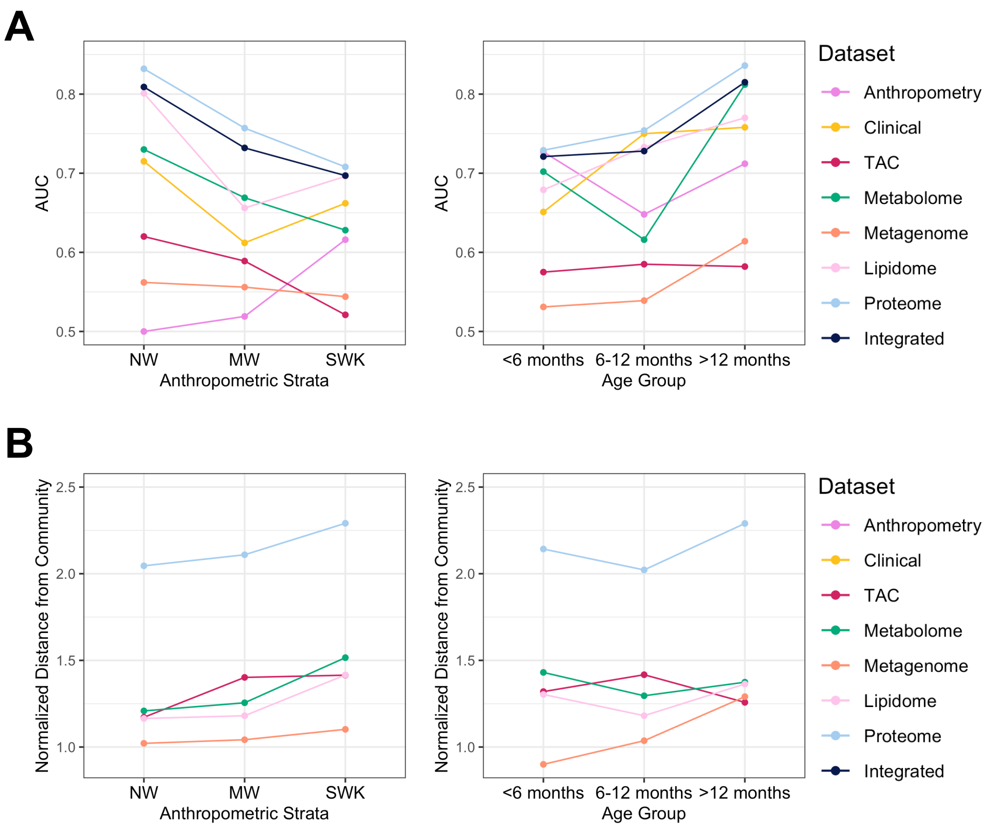

Figure S21. Characterizing the impact of anthropometric strata and age on the multiomic signature of mortality at discharge. Cross-validated XGBoost models for the prediction of mortality in the post-discharge period of the discovery cohort were trained on the data collected at discharge. (**A**) Model performances stratified by anthropometric strata (left) and age group (right). (**B**) Median normalized distance from the community stratified by anthropometric strata and age group for each omic dataset analyzed and the integrated multiomic data. NW = Not Wasted, MW = Moderately Wasted, and SWK = Severely Wasted or Kwashiorkor.

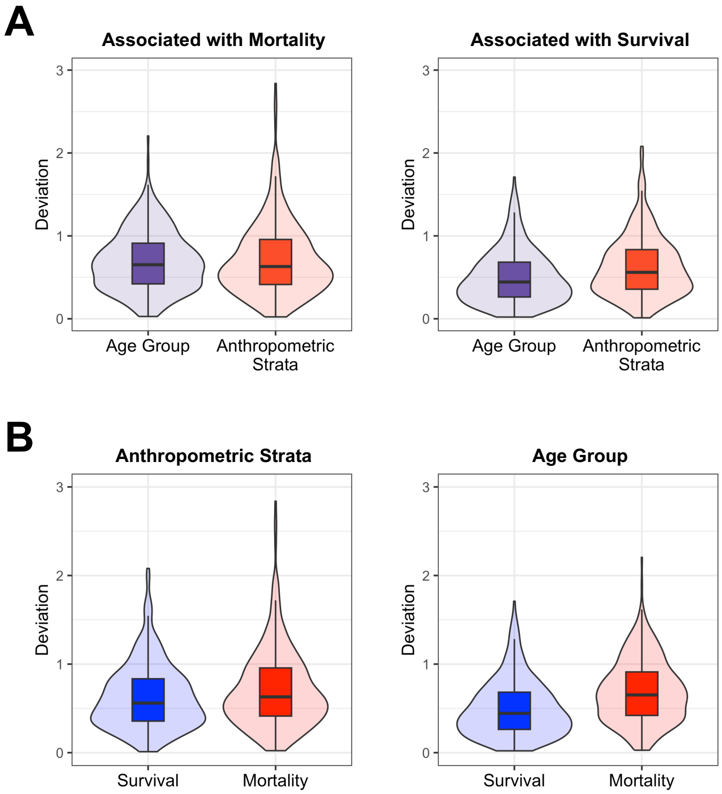

Figure S22. Feature associations with mortality and survival within anthropometric strata and age groups at discharge. Feature deviation scores within anthropometric strata and age groups were calculated at discharge for each feature significantly associated with mortality or survival with at least a small effect size (|hedge's g| > 0.2 (**A**) The distribution of feature deviation scores at discharge by association with mortality (left) or survival (right). (**B**) The distribution of feature deviation scores at discharge by anthropometric strata (left) and age group (right). Box plots indicate median (middle line); 25th and 75th percentiles (box limits); 1.5*interquartile range (error bars); and outliers (single points).

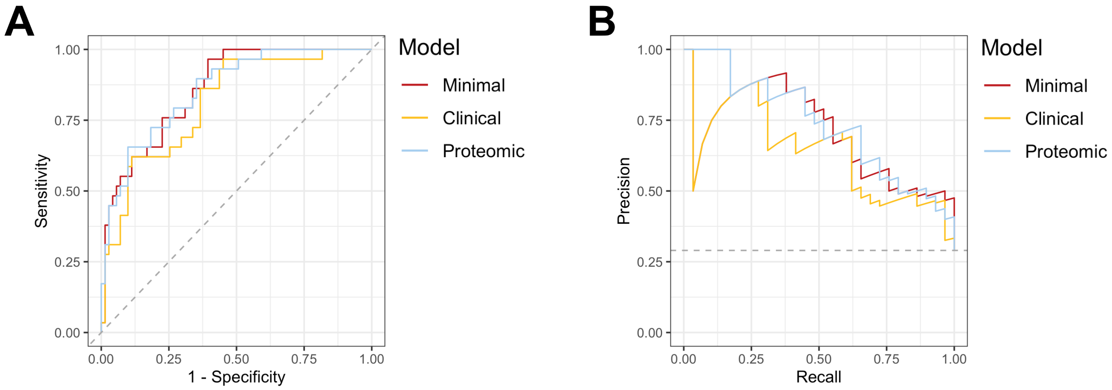

Figure S23. Performance on the validation cohort of the minimal model for the prediction of mortality during hospitalization and post-discharge. The minimal, proteomic, and clinical XGBoost model for the prediction of mortality trained on the discovery cohort were applied on the validation cohort. ROC (**A**) and precision-recall (**B**) curves for the minimal, proteomic, and clinical models.

A

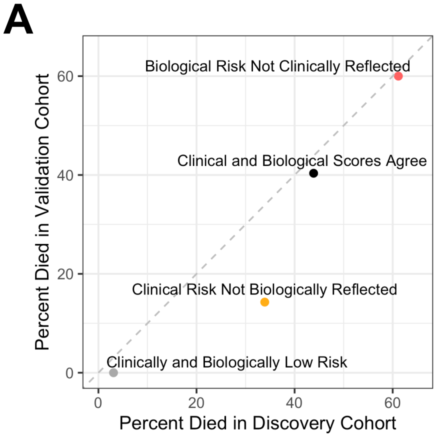

Figure S24. Characterization of the patient subgroups with discrepant clinical and multiomic mortality risk scores in the validation cohort. The cross-validated XGBoost models for the prediction of mortality during hospitalization or in the post-discharge period trained on the proteomics data and the clinical data of the discovery cohort were used to generate mortality risk scores for the validation cohort. (**A**) Comparison of subgroup percent mortality in the discovery cohort and the validation cohort for the patient subgroups defined in **Figure 3A** and **Figure 6G**.

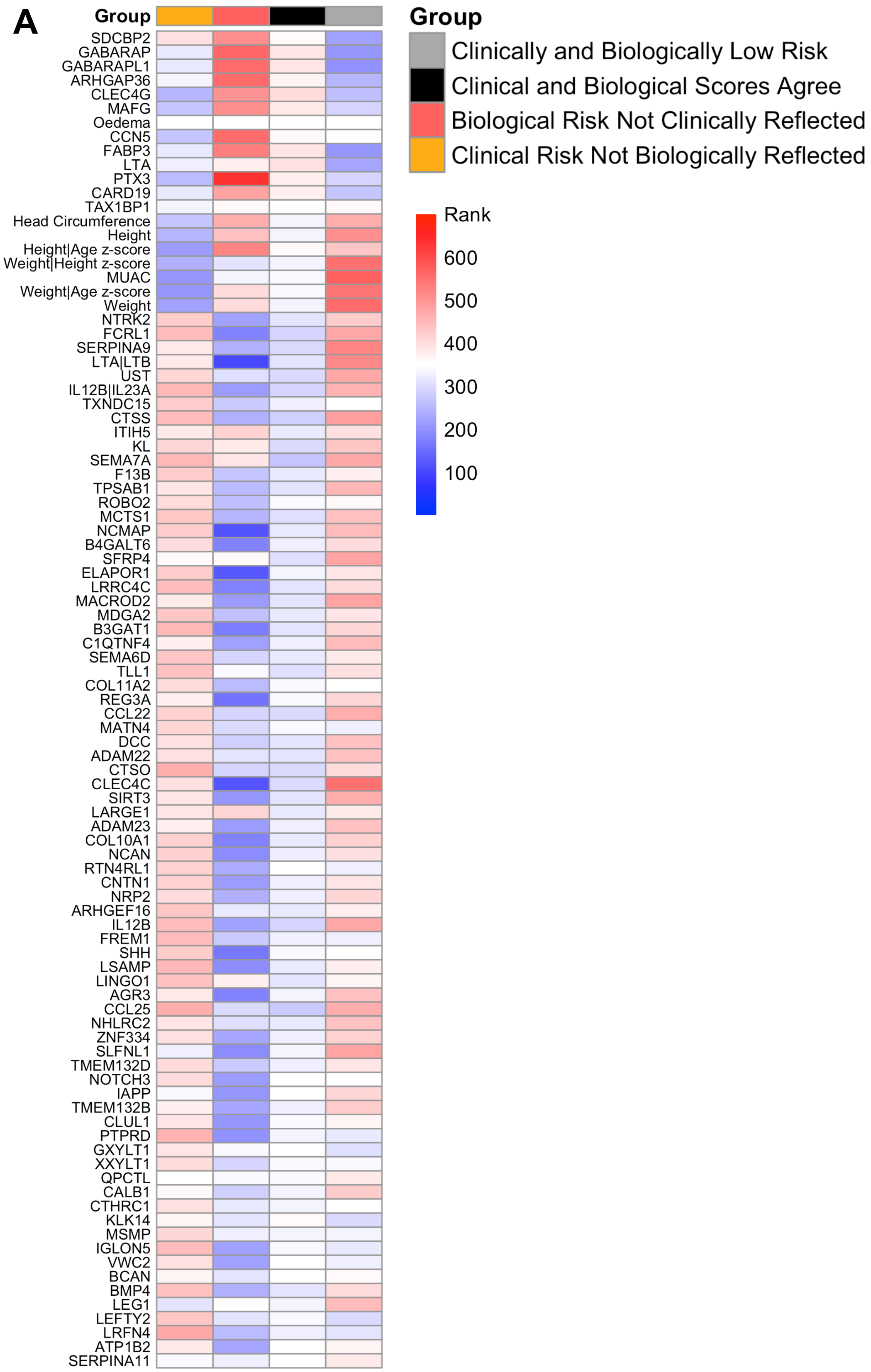
A
Group
SDCBP2
GABARAP
GABARAPL1
ARHGAP36
CLEC4G
MAFG
Oedema
CCN5
FABP3
LTA
PTX3
CARD19
TAX1BP1
Head Circumference
Height
Height|Age z-score
Weight|Height z-score
MUAC
Weight|Age z-score
Weight
NTRK2
FCRL1
SERPINA9
LTA|LTB
UST
IL12B|IL23A
TXNDC15
CTSS
ITIH5
KL
SEMA7A
F13B
TPSAB1
ROBO2
MCTS1
NCMAP
B4GALT6
SFRP4
ELAPOR1
LRRC4C
MACROD2
MDGA2
B3GAT1
C1QTNF4
SEMA6D
TLL1
COL11A2
REG3A
CCL22
MATN4
DCC
ADAM22
CTSO
CLEC4C
SIRT3
LARGE1
ADAM23
COL10A1
NCAN
RTN4RL1
CNTN1
NRP2
ARHGEF16
IL12B
FREM1
SHH
LSAMP
LINGO1
AGR3
CCL25
NHLRC2
ZNF334
SLFNL1
TMEM132D
NOTCH3
IAPP
TMEM132B
CLUL1
PTPRD
GXYLT1
XXYLT1
QPCTL
CALB1
CTHRC1
KLK14
MSMP
IGLON5
VWC2
BCAN
BMP4
LEG1
LEFTY2
LRFN4
ATP1B2
SERPINA11
Group
Clinically and Biologically Low Risk
Clinical and Biological Scores Agree
Biological Risk Not Clinically Reflected
Clinical Risk Not Biologically Reflected
Rank
600
500
400
300
200
100

Figure S25. Contrasting features of the patient subgroups with discrepant clinical and multiomic mortality risk scores in the validation cohort. The cross-validated XGBoost models for the prediction of mortality during hospitalization or in the post-discharge period trained on the proteomics data and the clinical data of the discovery cohort were used to generate mortality risk scores for the validation cohort. (**A**) Heatmap of the clinical and multiomic features most different between the discrepant subgroups defined in **Figure 3A** assessed in the validation cohort.

Supplementary Tables

Table S1: Features driving the predictive power of the multiomic admissions model for the prediction of mortality during hospitalization and post-discharge.

Dataset	Feature	Name
Lipidome	TG_(58:10)	TG 58:10
Proteome	seq.10743.13	SLITRK1
Proteome	seq.13118.5	SMOC1
Proteome	seq.15295.81	IGF2
Proteome	seq.2571.12	IGFBP3
Proteome	seq.2952.75	IGF1
Proteome	seq.3291.30	FCER2
Proteome	seq.5437.63	FABP3
Proteome	seq.5728.60	FCRL1
Proteome	seq.9094.5	CLEC4C
Proteome	seq.4234.8	IL1RL1
Proteome	seq.8235.48	CHGB

Table S2: Performance statistics of the models at hospital admission built on each dataset for the prediction of mortality during hospitalization or in the post-discharge period. Significance was assessed using a two-sided Wilcoxon rank-sum test.

**Dataset**	***N***	**AUROC [95% CI]**	**AUPRC [95% CI]**	**Wilcoxon *P***
Anthropometry	1001	0.73 [0.70, 0.76]	0.56 [0.51, 0.61]	$2.5*10^{-32}$
Clinical Variables	966	0.80 [0.77, 0.83]	0.70 [0.65, 0.74]	$3.6*10^{-51}$
TAC	993	0.66 [0.63, 0.70]	0.50 [0.46, 0.55]	$6.5*10^{-17}$
Metabolome	924	0.80 [0.77, 0.83]	0.69 [0.64, 0.73]	$6.0*10^{-51}$
Metagenome	757	0.62 [0.57, 0.66]	0.46 [0.41, 0.51]	$1.7*10^{-7}$
Lipidome	906	0.82 [0.79, 0.85]	0.69 [0.64, 0.74]	$3.8*10^{-55}$
Proteome	945	0.85 [0.83, 0.88]	0.76 [0.72, 0.80]	$1.8*10^{-69}$
Multiomic Integrated	1007	0.84 [0.82, 0.87]	0.75 [0.71, 0.79]	$6.7*10^{-72}$

Table S3: Performance statistics of the multiomic model at hospital admission for the prediction of mortality during hospitalization or in the post-discharge period stratified by study site. Significance was assessed using a two-sided Wilcoxon rank-sum test.

**Study Site**	***N***	**Percent Died**	**AUROC [95% CI]**	**AUPRC [95% CI]**	**Wilcoxon *P***
Banfora	141	39.0%	0.80 [0.72, 0.87]	0.75 [0.65, 0.85]	$3.0*10^{-9}$
Blantyre	113	34.5%	0.86 [0.78, 0.93]	0.76 [0.64, 0.88]	$5.2*10^{-10}$
Dhaka	108	18.5%	0.81 [0.71, 0.92]	0.51 [0.33, 0.71]	$1.3*10^{-5}$
Kampala	155	34.2%	0.79 [0.71, 0.86]	0.67 [0.56, 0.80]	$5.4*10^{-9}$
Karachi	110	32.7%	0.79 [0.70, 0.87]	0.66 [0.53, 0.78]	$1.2*10^{-6}$
Kilifi	74	29.7%	0.93 [0.88, 0.99]	0.86 [0.74, 0.95]	$5.0*10^{-9}$
Matlab	79	8.9%	0.85 [0.62, 1.00]	0.71 [0.40, 0.98]	$2.6*10^{-3}$
Mbagathi	102	44.1%	0.78 [0.69, 0.87]	0.78 [0.68, 0.86]	$1.2*10^{-6}$
Migori	125	58.4%	0.81 [0.74, 0.89]	0.85 [0.77, 0.91]	$2.6*10^{-9}$

Table S4: Features of the minimal admissions model for the prediction of mortality during hospitalization and post-discharge.

Dataset	Feature	Name
Lipidome	TG_(58:10)	TG 58:10
Proteome	seq.10743.13	SLITRK1
Proteome	seq.13118.5	SMOC1
Proteome	seq.22560.1	EVI2A
Proteome	seq.2571.12	IGFBP3
Proteome	seq.3069.52	IGHM
Proteome	seq.3505.6	LTA|LTB
Proteome	seq.5437.63	FABP3
Proteome	seq.5728.60	FCRL1
Proteome	seq.9094.5	CLEC4C

Table S5: Features of the minimal admissions model for the classification of patients into discrepant subgroups.

Dataset	Feature	Name
Lipidome	PE_(40:7)	PE 40:7
Proteome	seq.10435.2	AGA
Proteome	seq.13618.15	MAD1L1
Proteome	seq.15653.9	COL10A1
Proteome	seq.22985.160	IGFBP2
Proteome	seq.23591.9	EXD1
Proteome	seq.25960.15	PLS3
Proteome	seq.5699.19	FAM189A2
Proteome	seq.6383.90	TLL1
Proteome	seq.9185.15	TFF1

Table S6: Performance statistics of the models built on each dataset at discharge for the prediction of mortality in the post-discharge period. Significance was assessed using a two-sided Wilcoxon rank-sum test.

Dataset	*N*	AUROC [95% CI]	AUPRC [95% CI]	Wilcoxon *P*
Anthropometry	825	0.70 [0.66, 0.75]	0.40 [0.34, 0.47]	$3.2*10^{-16}$
Clinical Variables	802	0.72 [0.68, 0.77]	0.40 [0.34, 0.47]	$1.6*10^{-18}$
TAC	739	0.58 [0.53, 0.64]	0.24 [0.20, 0.30]	0.003
Metabolome	648	0.70 [0.64, 0.75]	0.37 [0.30, 0.46]	$3.2*10^{-11}$
Metagenome	575	0.53 [0.47, 0.59]	0.21 [0.17, 0.26]	0.34
Lipidome	657	0.73 [0.68, 0.78]	0.37 [0.31, 0.45]	$3.6*10^{-15}$
Proteome	681	0.78 [0.73, 0.82]	0.44 [0.37, 0.53]	$1.9*10^{-21}$
Multiomic Integrated	750	0.77 [0.72, 0.81]	0.44 [0.37, 0.51]	$1.5*10^{-22}$

Table S7: Performance statistics of the multiomic model at discharge for the prediction of mortality in the post-discharge period stratified by study site. Significance was assessed using a two-sided Wilcoxon rank-sum test.

**Study Site**	***N***	**Percent Died**	**AUROC [95% CI]**	**AUPRC [95% CI]**	**Wilcoxon *P***
Banfora	103	19.4%	0.67 [0.54, 0.80]	0.31 [0.21, 0.51]	0.02
Blantyre	74	21.6%	0.81 [0.70, 0.91]	0.56 [0.37, 0.75]	$2.1*10^{-4}$
Dhaka	99	11.1%	0.77 [0.60, 0.94]	0.33 [0.16, 0.58]	0.004
Kampala	108	13.0%	0.70 [0.56, 0.83]	0.33 [0.15, 0.57]	0.02
Karachi	85	27.1%	0.73 [0.61, 0.86]	0.58 [0.41, 0.74]	0.001
Kilifi	58	15.5%	0.81 [0.68, 0.94]	0.46 [0.23, 0.76]	0.002
Matlab	74	5.4%	0.97 [0.91, 1.00]	0.75 [0.34, 1.00]	0.002
Mbagathi	75	26.7%	0.75 [0.61, 0.89]	0.57 [0.38, 0.78]	0.001
Migori	74	31.1%	0.60 [0.47, 0.74]	0.36 [0.28, 0.54]	0.16

Table S8: Normalized omic distance comparisons between anthropometric strata and age groups at hospital admission. Significance was assessed using a two-sided Wilcoxon rank-sum test. Dataset and comparisons not present did not achieve FDR-corrected statistical significance. NW = Not Wasted, MW = Moderately Wasted, and SWK = Severely Wasted or Kwashiorkor. FDR = False Discovery Rate.

**Dataset**	**Groups Compared**		**FDR-corrected Wilcoxon *P***
TAC	<6 months	6-12 months	0.001
Metagenome	6-12 months	>12 months	0.02
Lipidome	6-12 months	>12 months	0.01
Proteome	<6 months	>12 months	0.02
TAC	NW	MW	0.03
TAC	NW	SWK	$5.3*10^{-4}$
Metabolome	NW	SWK	$4.6*10^{-7}$
Metabolome	MW	SWK	0.01
Lipidome	NW	SWK	$4.6*10^{-7}$
Lipidome	MW	SWK	$6.5*10^{-6}$
Proteome	NW	SWK	$1.4*10^{-6}$
Proteome	MW	SWK	$1.9*10^{-4}$

Table S9: Normalized omic distance comparisons between anthropometric strata and age groups at discharge. Significance was assessed using a two-sided Wilcoxon rank-sum test. Dataset and comparisons not present did not achieve FDR-corrected statistical significance. NW = Not Wasted, MW = Moderately Wasted, and SWK = Severely Wasted or Kwashiorkor. FDR = False Discovery Rate.

**Dataset**	**Groups Compared**		**FDR-corrected Wilcoxon *P***
Metagenome	<6 months	6-12 months	0.03
Metagenome	<6 months	>12 months	$4.0*10^{-5}$
Metagenome	6-12 months	>12 months	0.03
Metabolome	NW	SWK	$2.8*10^{-9}$
Metabolome	MW	SWK	$1.4*10^{-5}$
Lipidome	NW	SWK	$9.2*10^{-4}$
Lipidome	MW	SWK	0.005
Proteome	NW	SWK	0.007

Table S10: Features of the minimal model for the prediction of mortality on the validation cohort.

Dataset	Feature	Name
Proteome	seq.10743.13	SLITRK1
Proteome	seq.10781.19	CLEC4G
Proteome	seq.13118.5	SMOC1
Proteome	seq.15295.81	IGF2
Proteome	seq.2571.12	IGFBP3
Proteome	seq.2952.75	IGF1
Proteome	seq.4234.8	IL1RL1
Proteome	seq.5437.63	FABP3
Proteome	seq.5728.60	FCRL1
Proteome	seq.6557.50	LRRC15

Table S11: Features of the minimal model for the classification of patients into discrepant subgroups in the validation cohort.

Dataset	Feature	Name
Proteome	seq.13104.32	EFNB1
Proteome	seq.13658.31	PDGFC
Proteome	seq.13979.3	SLC26A7
Proteome	seq.16919.1	DBI
Proteome	seq.23632.22	SLFNL1
Proteome	seq.3505.6	LTA|LTB
Proteome	seq.7266.4	SERPINA9
Proteome	seq.8445.184	MELT
Proteome	seq.9094.5	CLEC4C
