# Supplementary material for: Multiomics characterization of acute child illness and mortality in Africa and South Asia
Source: Nat Commun. 2026 Apr 13;17:5171. doi: 10.1038/s41467-026-69754-w (PMC13249890; doi:10.1038/s41467-026-69754-w)
Supplement: Supplementary file 2 — Reporting Summary [file 41467_2026_69754_MOESM2_ESM.pdf]

nature portfolio

Corresponding author(s): Judd Walson
Jay Berkley
Nima Aghaeepour

Last updated by author(s): Dec 15, 2025

Reporting Summary

Nature Portfolio wishes to improve the reproducibility of the work that we publish. This form provides structure for consistency and transparency in reporting. For further information on Nature Portfolio policies, see our Editorial Policies and the Editorial Policy Checklist.

Statistics

For all statistical analyses, confirm that the following items are present in the figure legend, table legend, main text, or Methods section.

n/a	Confirmed	
☐	☒	The exact sample size (*n*) for each experimental group/condition, given as a discrete number and unit of measurement
☒	☐	A statement on whether measurements were taken from distinct samples or whether the same sample was measured repeatedly
☐	☒	The statistical test(s) used AND whether they are one- or two-sided<br>*Only common tests should be described solely by name; describe more complex techniques in the Methods section.*
☐	☒	A description of all covariates tested
☐	☒	A description of any assumptions or corrections, such as tests of normality and adjustment for multiple comparisons
☐	☒	A full description of the statistical parameters including central tendency (e.g. means) or other basic estimates (e.g. regression coefficient) AND variation (e.g. standard deviation) or associated estimates of uncertainty (e.g. confidence intervals)
☐	☒	For null hypothesis testing, the test statistic (e.g. *F*, *t*, *r*) with confidence intervals, effect sizes, degrees of freedom and *P* value noted<br>*Give P values as exact values whenever suitable.*
☒	☐	For Bayesian analysis, information on the choice of priors and Markov chain Monte Carlo settings
☒	☐	For hierarchical and complex designs, identification of the appropriate level for tests and full reporting of outcomes
☐	☒	Estimates of effect sizes (e.g. Cohen's *d*, Pearson's *r*), indicating how they were calculated

Our web collection on statistics for biologists contains articles on many of the points above.

Software and code

Policy information about availability of computer code

Data collection

No code was used for data collection

Data analysis

All analyses were performed with R (Version 4.3.1) and all custom code is provided to reproduce the analyses in this study at https://doi.org/10.5061/dryad.p2ngf1w1z

Code versions are outlined below:
Package Version
AnnotationDbi 1.64.1
BH 1.87.0-1
Biobase 2.62.0
BiocBaseUtils 1.4.0
BiocGenerics 0.48.1
BiocManager 1.30.26
BiocParallel 1.36.0
BiocVersion 3.17.1
Biostrings 2.70.3
DBI 1.2.3
DEoptimR 1.1-4
DelayedArray 0.28.0
Formula 1.2-5
GO.db 3.17.0

GenomeInfoDb 1.38.8
GenomeInfoDbData 1.2.11
GenomicRanges 1.54.1
Hmisc 5.2-4
IRanges 2.36.0
KEGGREST 1.42.0
KernSmooth 2.23-26
MASS 7.3-60
Matrix 1.6-5
MatrixGenerics 1.14.0
ModelMetrics 1.2.2.2
MultiAssayExperiment 1.28.0
MultiDataSet 1.30.0
PRROC 1.4
R6 2.6.1
RColorBrewer 1.1-3
RCurl 1.98-1.17
RSQLite 2.4.3
Rcpp 1.1.0
RcppArmadillo 15.0.2-2
RcppEigen 0.3.4.0.2
Rdpack 2.6.4
S4Arrays 1.2.1
S4Vectors 0.40.2
SQUAREM 2021.1
SparseArray 1.2.4
SparseM 1.84-2
SummarizedExperiment 1.32.0
XVector 0.42.0
abind 1.4-8
askpass 1.2.1
backports 1.5.0
base64enc 0.1-3
bayesm 3.1-6
bit 4.6.0
bit64 4.6.0-1
bitops 1.0-9
blob 1.2.4
boot 1.3-31
bslib 0.9.0
cachem 1.1.0
calibrate 1.7.7
caret 7.0-1
checkmate 2.3.3
class 7.3-23
cli 3.6.5
clock 0.7.3
cluster 2.1.8.1
codetools 0.2-20
colorspace 2.1-2
compositions 2.0-9
cowplot 1.2.0
cpp11 0.5.2
crayon 1.5.3
crosstalk 1.2.2
curl 7.0.0
data.table 1.17.8
diagram 1.6.5
digest 0.6.37
dplyr 1.1.4
e1071 1.7-16
effsize 0.8.1
evaluate 1.0.5
farver 2.1.2
fastmap 1.2.0
fastmatch 1.1-6
fgsea 1.28.0
fontawesome 0.5.3
forcats 1.0.1
foreach 1.5.2
foreign 0.8-90
formatR 1.14
fs 1.6.6
futile.logger 1.4.3
futile.options 1.0.1
future 1.67.0

future.apply 1.20.0
generics 0.1.4
ggalluvial 0.12.5
ggplot2 3.5.2
ggrepel 0.9.6
ggtern 3.5.0
globals 0.18.0
glue 1.8.0
gmm 1.9-1
gower 1.0.2
graph 1.80.0
gridExtra 2.3
gtable 0.3.6
hardhat 1.4.2
hexbin 1.28.5
highr 0.11
htmlTable 2.4.3
htmltools 0.5.8.1
htmlwidgets 1.6.4
httr 1.4.7
impute 1.76.0
imputeLCMD 2.1
ipred 0.9-15
isoband 0.2.7
iterators 1.0.14
jquerylib 0.1.4
jsonlite 2.0.0
knitr 1.5
labeling 0.4.3
lambda.r 1.2.4
later 1.4.4
latex2exp 0.9.6
lattice 0.22-7
lava 1.8.2
lazyeval 0.2.2
lifecycle 1.0.4
limma 3.58.1
lipidr 2.14.1
listenv 0.9.1
lme4 1.1-37
lpSolve 5.6.23
lubridate 1.9.4
magrittr 2.0.4
mathjaxr 1.8-0
matrixStats 1.5.0
mediation 4.5.1
memoise 2.0.1
metadat 1.4-0
metafor 4.8-0
mgcv 1.9-1
mime 0.13
minqa 1.2.8
mvtnorm 1.3-3
nlme 3.1-168
nloptr 2.2.1
nnet 7.3-20
norm 1.0-11.1
numDeriv 2016.8-1.1
openssl 2.3.4
org.Hs.eg.db 3.17.0
pROC 1.19.0.1
parallelly 1.45.1
pbapply 1.7-4
pcaMethods 1.94.0
pheatmap 1.0.13
pillar 1.11.1
pkgconfig 2.0.3
plogr 0.2.0
plotly 4.11.0
plyr 1.8.9
png 0.1-8
prodlim 2025.04.28
progressr 0.17.0
promises 1.3.3
proto 1.0.0
proxy 0.4-27

purrr 1.1.0
qqman 0.1.9
rappdirs 0.3.3
rbibutils 2.3
recipes 1.3.1
reformulas 0.4.2
renv 1.1.5
reshape2 1.4.4
rlang 1.1.6
rmarkdown 2.3
robustbase 0.99-6
ropls 1.34.0
rpart 4.1.24
rstudioapi 0.17.1
sandwich 3.1-1
sass 0.4.10
scales 1.4.0
shape 1.4.6.1
snow 0.4-4
sparsevctrs 0.3.4
statmod 1.5.1
stringi 1.8.7
stringr 1.5.2
survival 3.8-3
sys 3.4.3
tensorA 0.36.2.1
tibble 3.3.0
tidyr 1.3.1
tidyselect 1.2.1
timeDate 4051.111
timechange 0.3.0
tinytex 0.57
tmvtnorm 1.7
topGO 2.52.0
tzdb 0.5.0
utf8 1.2.6
vctrs 0.6.5
viridisLite 0.4.2
withr 3.0.2
xfun 0.52
xgboost 1.7.11.1
yaml 2.3.10
zlibbioc 1.48.2
zoo 1.8-14

For manuscripts utilizing custom algorithms or software that are central to the research but not yet described in published literature, software must be made available to editors and reviewers. We strongly encourage code deposition in a community repository (e.g. GitHub). See the Nature Portfolio guidelines for submitting code & software for further information.

Data

Policy information about availability of data

All manuscripts must include a data availability statement. This statement should provide the following information, where applicable:

- Accession codes, unique identifiers, or web links for publicly available datasets
- A description of any restrictions on data availability
- For clinical datasets or third party data, please ensure that the statement adheres to our policy

All data used for this study is available at https://doi.org/10.7910/DVN/X6FAGX

Research involving human participants, their data, or biological material

Policy information about studies with human participants or human data. See also policy information about sex, gender (identity/presentation), and sexual orientation and race, ethnicity and racism.

Reporting on sex and gender	Assigned sex was used as a clinical covariate in the data collection. Assigned sex was used since all participants are under 24 months of age. No differences were found in the results when stratifying by assigned sex.
Reporting on race, ethnicity, or other socially relevant groupings	Race was not used as a stratifying variable in our study, but geographical location was given the highly diverse nature of our multinational cohort. The impact of location of origin on the results of the study are discussed in the manuscript.
Population characteristics	Participants were infants under 24 months of age across 9 sites in 6 different countries which were hospitalized at time of enrollment.

Recruitment	Briefly, 3101 acutely ill children were recruited from nine sites in six countries in sub-Saharan Africa and South Asia. Children were enrolled at admission to hospital and followed up for 180 days after discharge from hospital. Participating sites included rural and urban hospitals in Bangladesh (icddr,b Dhaka Hospital and Matlab Hospital), Burkina Faso (Banfora Regional Referral Hospital), Kenya (Kilifi County Hospital, Mbagathi Sub-County Hospital and Migori County Referral Hospital), Malawi (Queen Elizabeth Central Hospital), Pakistan (Civil Hospital, Karachi), and Uganda (Mulago Hospital). The mid-upper arm circumference (MUAC) was used to enrol children into three strata that included not wasted (MUAC ≥12.5cm for age ≥six months or MUAC ≥12cm for age <six months), moderately wasted (MUAC 11.5 to <12.5 cm for age ≥six months or MUAC 11 to <12 cm for age <six months), and severely wasted or kwashiorkor (oedematous malnutrition) (MUAC <11.5cm for age ≥six months or MUAC <11cm for age <six months or bilateral pedal oedema unexplained by other medical causes). In addition, children in the same communities as hospitalised cases who were not acutely ill: community participants (CP, N=1140) were recruited at a single time-point to establish community norms for demographic and biological factors. Community children 57 were included into the study if they had no hospital admission in the 14 days prior to contact with the study team and were not ill and therefore are considered to be well and typical for the community rather than 'healthy'. These community children were from the same neighbourhoods as acutely ill children and therefore, may have had anthropometric deficits, micronutrient deficiencies, helminth infections among others and therefore would not be regarded as 'healthy'.
Ethics oversight	The study protocol was reviewed and approved by the Oxford Tropical Research Ethics Committee, UK; the Kenya Medical Research Institute, Kenya; the University of Washington and Oregon Health and Science University, USA; Makerere University School of Biomedical Sciences Research Ethics Committee and The Uganda National Council for Science and Technology, Uganda; Aga Khan University, Pakistan; the International Centre for Diarrhoeal Disease Research, Bangladesh; The University of Malawi; The University of Ouagadougou and Centre Muraz, Burkina Faso; the Hospital for Sick Children, Canada; and University of Amsterdam, The Netherlands.

Note that full information on the approval of the study protocol must also be provided in the manuscript.

Field-specific reporting

Please select the one below that is the best fit for your research. If you are not sure, read the appropriate sections before making your selection.

☒ Life sciences ☐ Behavioural & social sciences ☐ Ecological, evolutionary & environmental sciences

For a reference copy of the document with all sections, see nature.com/documents/nr-reporting-summary-flat.pdf

Life sciences study design

All studies must disclose on these points even when the disclosure is negative.

Sample size	A nested case-cohort design drawing from a larger cohort provides estimates that are representative of the entire cohort while also yielding slightly more statistical power than a case control design 58 . Case-cohort selection involves a random subsample of the original cohort (sub-cohort), independently of how cases are defined, followed by additional inclusion of all cases outside the sub-cohort creating the case-cohort set 59 . This results in overrepresentation of the cases in the case-cohort set compared to the original cohort which is dealt with in analysis by weighting. A further advantage of this design over a case-control design is that the sub-cohort can also be used in analysis of other outcomes. In CHAIN, this could include outcomes of readmission or growth among survivors in future analyses, as well comparisons between acutely ill children and community children and between nutritional strata among acutely ill children in the current analysis.<br><br>The original CHAIN cohort stratification was maintained in the CNCC design; thus, a random 24% sub-cohort of children enrolled in the CHAIN cohort stratified by site was selected. This included 658 survivors (sub-cohort non-cases) and 109 deaths (sub-cohort cases). Thereafter, all remaining deaths (n=241 cases) that were not selected in the random 24% sub-cohort (Figure 3) were added, giving a total of 350 cases. An additional 30 randomly selected community participants from each site (total N=270) were chosen.<br><br>Multiple studies in both low- and high-income settings indicate a prolonged increased mortality risk after admission (including >1 year) compared to the community peers 10, 43, 60 which is now considered a well-recognised phenomenon. The primary analyses will be of acute mortality occurring within 30-days from admission and post-discharge mortality within 180-days from discharge using samples collected at admission and discharge timepoints. Power and effect size are a function of the number of cases, correlation between cases and survivors, and the ratio of cases to survivors. It is likely that the strength of association between exposures of interests and death will vary in magnitude between the 30-day and 180-day mortality periods. Sample size calculations suggest that a ratio of one case to two non-cases is adequate to reliably detect an HR of 1.5 at >80% power. The design selected all available deaths and simulation-based power calculations suggested that adding more non-cases per cases would yield minimal gains in power while incurring substantial additional expense.
Data exclusions	No data were excluded
Replication	Samples were analyzed once to generate each omic dataset. There were no replications or independent replicates.
Randomization	Randomization was not relevant for this study given that all participants received the same treatments and groupings were made based on patient outcomes
Blinding	Blinding was not relevant to the study given that all participants received the same treatments and groupings were made based on patient outcomes.

Reporting for specific materials, systems and methods

We require information from authors about some types of materials, experimental systems and methods used in many studies. Here, indicate whether each material, system or method listed is relevant to your study. If you are not sure if a list item applies to your research, read the appropriate section before selecting a response.

Materials & experimental systems

n/a	Involved in the study
☒	☐ Antibodies
☒	☐ Eukaryotic cell lines
☒	☐ Palaeontology and archaeology
☒	☐ Animals and other organisms
☒	☐ Clinical data
☒	☐ Dual use research of concern
☒	☐ Plants

Methods

n/a	Involved in the study
☒	☐ ChIP-seq
☒	☐ Flow cytometry
☒	☐ MRI-based neuroimaging

Plants

Seed stocks	*Report on the source of all seed stocks or other plant material used. If applicable, state the seed stock centre and catalogue number. If plant specimens were collected from the field, describe the collection location, date and sampling procedures.*
Novel plant genotypes	*Describe the methods by which all novel plant genotypes were produced. This includes those generated by transgenic approaches, gene editing, chemical/radiation-based mutagenesis and hybridization. For transgenic lines, describe the transformation method, the number of independent lines analyzed and the generation upon which experiments were performed. For gene-edited lines, describe the editor used, the endogenous sequence targeted for editing, the targeting guide RNA sequence (if applicable) and how the editor was applied.*
Authentication	*Describe any authentication procedures for each seed stock used or novel genotype generated. Describe any experiments used to assess the effect of a mutation and, where applicable, how potential secondary effects (e.g. second site T-DNA insertions, mosiacism, off-target gene editing) were examined.*
