# Supplementary material for: Multiomics characterization of acute child illness and mortality in Africa and South Asia
Source: Nat Commun. 2026 Apr 13;17:5171. doi: 10.1038/s41467-026-69754-w (PMC13249890; doi:10.1038/s41467-026-69754-w)
Supplement: Supplementary file 3 — Transparent Peer Review File [file 41467_2026_69754_MOESM3_ESM.pdf]

nature portfolio

Peer Review File

Multiomics characterization of acute child illness and mortality in Africa and South Asia

Corresponding Author: Professor Nima Aghaeepour

This manuscript has been previously reviewed at another journal. This document only contains information relating to versions considered at Nature Communications.

This file contains all reviewer reports in order by version, followed by all author rebuttals in order by version.

Version 0:

Reviewer comments:

Reviewer #1

(Remarks to the Author)
The authors have addressed all the main comments and their revisions have improved the manuscript.

(Remarks on code availability)
The authors provide comprehensive and well-documented R code for their manuscript.

Reviewer #2

(Remarks to the Author)
The authors have addressed my comments

(Remarks on code availability)
The authors have mentioned they have uploaded the code, however it's still not available to me. Please check the accuracy of the link:http://datadryad.org/stash/share/P1NC9xYPTGWlwf4dpvG76OUwv2pVJkHyZFIeDeUfqKc

Open Access This Peer Review File is licensed under a Creative Commons Attribution 4.0 International License, which permits use, sharing, adaptation, distribution and reproduction in any medium or format, as long as you give appropriate credit to the original author(s) and the source, provide a link to the Creative Commons license, and indicate if changes were made.
In cases where reviewers are anonymous, credit should be given to 'Anonymous Referee' and the source.
The images or other third party material in this Peer Review File are included in the article's Creative Commons license, unless indicated otherwise in a credit line to the material. If material is not included in the article's Creative Commons license and your intended use is not permitted by statutory regulation or exceeds the permitted use, you will need to obtain permission directly from the copyright holder.
To view a copy of this license, visit https://creativecommons.org/licenses/by/4.0/
